# Gastrointestinal Dysmotility Predisposes to Colitis through Regulation of Gut Microbial Composition and Linoleic Acid Metabolism

**DOI:** 10.1002/advs.202306297

**Published:** 2024-03-13

**Authors:** Youhua Zhang, Feifei Song, Muqing Yang, Chunqiu Chen, Jiaqu Cui, Mengyu Xing, Yuna Dai, Man Li, Yuan Cao, Ling Lu, Huiyuan Zhu, Ying Liu, Chunlian Ma, Qing Wei, Huanlong Qin, Jiyu Li

**Affiliations:** ^1^ Department of Pathology Shanghai Tenth People's Hospital, Tongji University School of Medicine Shanghai 200072 China; ^2^ Department of General Surgery Shanghai Tenth People's Hospital, Tongji University School of Medicine Shanghai 200072 China; ^3^ Diagnostic and Treatment Center for Refractory Diseases of Abdomen Surgery Shanghai Tenth People's Hospital, Tongji University School of Medicine Shanghai 200072 China; ^4^ Department of Colorectal Disease Shanghai Tenth People's Hospital, Tongji University School of Medicine Shanghai 200072 China; ^5^ Department of Gastrointestinal Surgery Shanghai Tenth People's Hospital, Tongji University School of Medicine Shanghai 200072 China; ^6^ Geriatric Cancer Center HuaDong Hospital Affiliated to Fudan University Shanghai 200040 China

**Keywords:** gut microbiota, gut motility, immune cell, inflammatory bowel disease, linoleic acid

## Abstract

Disrupted gastrointestinal (GI) motility is highly prevalent in patients with inflammatory bowel disease (IBD), but its potential causative role remains unknown. Herein, the role and the mechanism of impaired GI motility in colitis pathogenesis are investigated. Increased colonic mucosal inflammation is found in patients with chronic constipation (CC). Mice with GI dysmotility induced by genetic mutation or chemical insult exhibit increased susceptibility to colitis, dependent on the gut microbiota. GI dysmotility markedly decreases the abundance of *Lactobacillus animlalis* and increases the abundance of *Akkermansia muciniphila*. The reduction in *L. animlalis*, leads to the accumulation of linoleic acid due to compromised conversion to conjugated linoleic acid. The accumulation of linoleic acid inhibits Treg cell differentiation and increases colitis susceptibility via inducing macrophage infiltration and proinflammatory cytokine expression in macrophage. *Lactobacillus* and *A. muciniphila* abnormalities are also observed in CC and IBD patients, and mice receiving fecal microbiota from CC patients displayed an increased susceptibility to colitis. These findings suggest that GI dysmotility predisposes host to colitis development by modulating the composition of microbiota and facilitating linoleic acid accumulation. Targeted modulation of microbiota and linoleic acid metabolism may be promising to protect patients with motility disorder from intestinal inflammation.

## Graphical abstract

The diminished gastrointestinal motility led to a reduction in bacteria containing linoleic acid isomerase, specifically *Lactobacillus animalis*, which caused the accumulation of linoleic acid due to compromised conversion to conjugated linoleic acid. Consequently, linoleic acid induced macrophage infiltration and the expression of proinflammatory cytokines in macrophages, ultimately inhibiting the differentiation of Treg cells and increasing susceptibility to colitis.

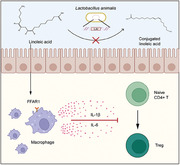

## 1 Introduction

Inflammatory bowel disease (IBD) is characterized by chronic and remitting inflammation of the gastrointestinal tract; IBD primarily includes two major clinical types: ulcerative colitis (UC) and Crohn's disease (CD). The prevalence of IBD is higher in developed Western countries, with up to 1.6 million Americans and 2 million Europeans affected by IBD,^[^ ^1^ ^]^ and is rapidly increasing in developing countries in South America, Asia, Africa, and Eastern Europe.^[^ ^1^, ^2^ ^]^ This trend suggests that environmental risk factors play critical roles in the development of IBD.^[^ ^3^ ^]^ Most environmental triggers, such as smoke and antibiotics, can mediate IBD pathogenesis through their impact on the microbiome.

Gastrointestinal (GI) motility is essential for gut homeostasis, which is maintained by a collaborative network formed by smooth muscle cells, enteric neurons, and interstitial cells of Cajal (ICC). It is generally thought that dysmotility is the consequence of IBD, however emerging evidence shows a possible pre‐occurrence of GI dysmotility prior to colitis onset. First, GI dysmotility is often seen before the presence of clinical manifestations of some diseases with intestinal inflammation such as necrotizing enterocolitis^[^ ^4^ ^]^ and Hirschsprung‐associated enterocolitis.^[^ ^5^ ^]^ Second, transgenic animals with altered numbers of enteric neurons due to the altered expression of *sox10*,^[^ ^6^ ^]^ Toll‐like receptor 2,^[^ ^7^ ^]^ *Hand2* or *noggin* ^[^ ^8^ ^]^ displayed severer or milder intestinal inflammation. Additionally, a recent study identified 51 protein biomarkers that were predictive of CD, including enteric nerve and muscle regulators such as neural cell adhesion molecule and erythropoietin receptor.^[^ ^9^ ^]^ This suggests that dysfunction of enteric nerves and muscles might occur before the onset of colitis. Third, transplantation of stem cells of ICC to mice before dextran sulfate sodium (DSS) administration reduced the severity of both acute and chronic colitis.^[^ ^10^ ^]^ Given that the transplanted stem cells of ICCs could cooperate to form a functional ICC network in vivo,^[^ ^11^ ^]^ this finding suggests that an integrated and functional ICC network is essential to prevent colitis development. Together, these findings suggests that an integral and functional GI motility network is vital to prevent colitis development, and dysmotility might occur prior to colitis onset and be a previously unrecognized trigger for IBD development.

GI motility is closely related with the richness and composition of the gut microbiota. A long colonic transit time, which indicates a possibly impaired motility, is positively correlated with species richness, negatively associated with the *Bacteroidetes*:*Firmicutes* ratio, and linked to *Akkermansia* and *Methanobrevibacter* abundance.^[^ ^12^ ^]^ Long colonic transit time is also accompanied by a shift in colonic metabolism from carbohydrate fermentation to protein catabolism.^[^ ^13^ ^]^ Treating mice with drugs that increase or decrease motility alters the composition and abundance of specific bacterial groups in the microbiota,^[^ ^14^ ^]^ which provide direct evidences that motility modulation could cause alterations in the microbiota. Moreover, microbial metabolites such as short‐chain fatty acids^[^ ^15^ ^]^ and serotonin can regulate GI motility by acting on smooth muscle cells and enteric neurons. These findings indicate a reciprocal interaction between the gut microbiota and motility; however, the resulting impact of such interactions on IBD pathogenesis remains unexplored.

ICCs are gut pacemaker cells located in the muscle layer throughout the GI tract and are indispensable in regulating GI musculature. The alteration of ICC number and function has been found to be related with the disrupted GI motility of various diseases including IBD.^[^ ^16^ ^]^ ICCs express the receptor tyrosine kinase c‐Kit and depend on c‐Kit‐stem cell factor signaling for their development. Thus, c‐Kit mutant animals can be used to investigate the function of ICC and GI musculature.^[^ ^17^ ^]^ Here, with c‐Kit receptor dysfunction‐based ICC‐deficient murine model (Kit^wsh/wsh^) and chemical‐induced murine model with decreased motility, together with clinical samples, we demonstrate that decreased GI motility might contribute to colitis development through modulating gut microbial composition (especially the abundance of *Lactobacillus* and *Akkermansia muciniphila*); and inducing linoleic acid accumulation which resulted in inhibited Treg cell differentiation in a macrophage‐dependent way.

## 2 Results

### 2.1 Increased Mucosal Inflammation in Colonic Tissue from Patients with Chronic Constipation

To determine the potential role of impaired GI motility in the pathogenesis of colitis, we first collected tissue samples from the normal colon region dissected from patients with colorectal cancer (CRC), colon region from patients with chronic constipation (CC), inflamed colon region from patients with CD and UC and examined inflammatory cytokine expression and immune cell infiltration in these four types of samples (See Table S1 in Supporting Information for patient characteristics). Inflamed samples from patients with CD and UC served as positive controls. We found that the expression of *IL1A*, *IL1B*, *CXCL8*, *and TNFA* was upregulated in colonic mucosa of patients with CC compared to that in normal controls (Figure S1A, Supporting Information). In addition, the infiltration of macrophages, CD4^+^ T cells and CD8^+^ T cells was increased in mucosa of patients with CC (Figure S1B,C, Supporting Information). These data demonstrate that the level of mucosal inflammation was increased in colonic mucosa in CC patients, suggesting that impaired gut motility is associated with increased inflammation.

### 2.2 GI Dysmotility Increased Susceptibility to DSS‐Induced Colitis

**Figure 1:**
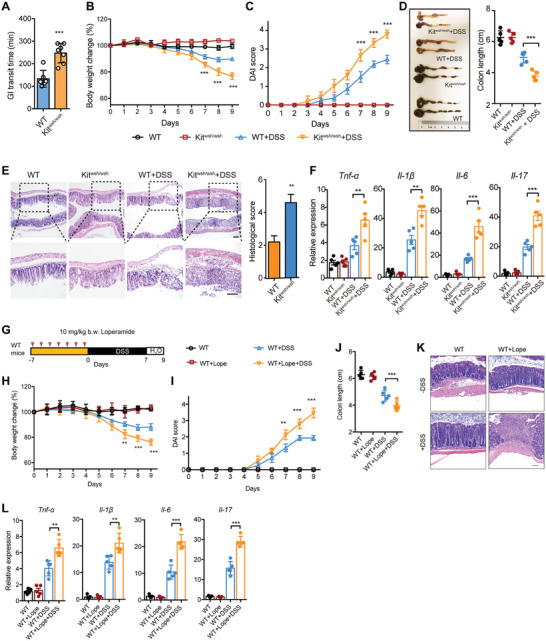
Interstitial cells of Cajal (ICC)‐deficient and loperamide induced‐GI dysmotility increased susceptivity to DSS‐induced colitis. A) The gut transit time of WT (n = 5) and Kit^wsh/wsh^ (n = 7) mice. B‐F) WT (n = 5) and Kit^wsh/wsh^ mice (n = 5) were treated with 2.5% DSS for 7 days, followed by water for 2 days. B) Body weight changes were monitored daily during DSS administration. C) Disease activity index (DAI) was monitored daily during DSS administration. D) Colon lengths were measured after mice were sacrificed on day 9. E) Colon tissues were examined histologically after H&E staining and scored for inflammation and architectural distortion. Scale bars, 50 µm. F) Inflammatory gene expression was examined by QPCR after mice were sacrificed on day 9. For loperamide (Lope) induced model G–L), WT mice (n = 5/group) were gavaged with 10 mg k^−1^g body weight (b.w.) loperamide every day for 7 days before DSS treatment to sacrifice G). The body weight changes H), DAI I) colon lengths J), representative images of H&E‐stained colon sections K), and colonic gene expression of proinflammatory genes L) in WT mice treated with or without loperamide were monitored or analyzed. Scale bar K), 100 µm. In A‐F, H‐J and L, data represent mean ± SEM; **P < 0.01, ***P < 0.001 by two‐sided Student *t* test.

To directly investigate whether impaired GI motility plays a role in colitis pathogenesis, we used DSS to induce colitis in both WT and Kit^wsh/wsh^ mice. Before DSS treatment, we first assessed the gut motility of WT and Kit^wsh/wsh^ mice. GI transit time was significantly increased in Kit^wsh/wsh^ mice, indicating impaired gut motility (**Figure** **1**A). After 7 days of DSS treatment, Kit^wsh/wsh^ mice exhibited increased weight loss compared to WT mice (Figure 1B), which was accompanied by a higher disease activity index (DAI), shorter colon length, increased histological score, and increased expression of proinflammatory cytokines (Figure 1C–F). We next examined the infiltration of T helper (Th) cell subsets Th1, Th17, and Treg in colonic lamina propria (cLP) of WT and Kit^wsh/wsh^ mice with or without DSS treatment. The ratio of Treg in CD4^+^ T cells decreased in Kit^wsh/wsh^ mice compared to that in WT mice, while the ratio of Th17 increased. This difference was further amplified by the administration of DSS treatment (Figure S2, Supporting Information).

To further confirm the effect of decreased GI motility on colitis development, we used another mouse model of impaired GI motility. We treated WT mice with loperamide for 7 days before the DSS treatment period to increase GI transit time (Figure 1G). To exclude the possibility that loperamide may potentially impact factors that affect susceptibility to colitis, we examined the integrity of gut epithelial in tissues from both control and loperamide‐treated mice prior to DSS challenge. QPCR and immunofluorescence analyses were performed to assess the expression of tight junction proteins in both ileal and colonic tissues from the two groups. The expression of *claudin‐1*, *claudin‐2*, junctional adhesion molecule A (*JAM‐A)*, *occludin* and zonula occludens 1 (*ZO‐1)* showed no significant differences between the tissues from the control and loperamide‐treated mice (Figure S3A, Supporting Information). Furthermore, immunofluorescence staining confirmed these results (Figure S3B, Supporting Information). Thus, these data indicate loperamide treatment could not induce any alterations in gut epithelial integrity. Mice pre‐treated with loperamide displayed increased susceptibility to DSS‐induced colitis, including increases in weight loss, DAI score, colon length shortening, and proinflammatory cytokine expression (Figure 1H–L). Together, these data indicate that GI dysmotility exacerbates the inflammatory response in the DSS‐induced colitis model.

### 2.3 GI Dysmotility‐Induced Colitis Susceptibility was Microbiota‐Dependent

**Figure 2:**
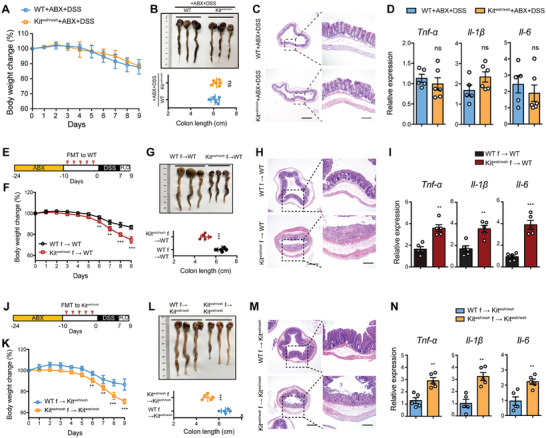
GI dysmotility‐induced colitis exacerbation is dependent on gut microbiota. A–D) WT (n = 5) and Kit^wsh/wsh^ (n = 6) mice were treated with a cocktail of antibiotics (ABX) for 14 days, and then treated with 2.5% DSS. The body weight changes A), colon lengths B), representative images of H&E‐stained colon sections C), and expression of proinflammatory genes D) were monitored or analyzed. E–I) WT mice (n = 5/group) were treated with a cocktail of ABX for 14 days, and then gavaged with fecal material from WT (WT f) or Kit^wsh/wsh^ (Kit^wsh/wsh^ f) mice every other day for 10 days, followed by a 2.5% DSS treatment for 7 days and water 2 days. The body weight changes F), colon lengths G), representative images of H&E‐stained colon sections H), and expression of proinflammatory genes I) in WT mice receiving fecal material from WT or Kit^wsh/wsh^ mice were monitored or analyzed. J–N) Kit^wsh/wsh^ mice (n = 5/group) were treated with a cocktail of ABX for 14 days, and then gavaged with fecal material from WT or Kit^wsh/wsh^ mice every other day for 10 days, followed by a 2.5% DSS treatment for 7 days and water 2 days. The body weight changes K), colon lengths L), representative images of H&E‐stained colon sections M), and colonic gene expression of proinflammatory genes N) in Kit^wsh/wsh^ mice receiving fecal material from WT or Kit^wsh/wsh^ mice were monitored or analyzed. Scale bars (C, H, M): left 500 µm, right 200 µm. In A, B, D, F, G, I, K, L, and N, data represent mean ± SEM; ns, not significant, **P < 0.01, ***P < 0.001 by two‐sided Student *t* test.

Next, we investigated the mechanism by which GI dysmotility exacerbates colitis development. Given that modulation of GI motility could alter the richness and composition of the gut microbiota that is closely related to colitis pathogenesis, we speculated that the gut microbiota may play a central role in motility‐regulated colitis development. To test our hypothesis, we first used a cocktail of antibiotics (ABX) containing ampicillin, streptomycin, and colistin to treat WT and Kit^wsh/wsh^ mice for 2 weeks to eliminate their gut microorganisms before DSS administration. Upon DSS treatment, antibiotic‐treated Kit^wsh/wsh^ mice and WT mice exhibited similar body weight change, colon length, colon morphology, and inflammatory cytokine expression (**Figure** **2**A–D), indicating that the gut microbiota might be involved in motility‐regulated colitis development. To directly determine whether the microbiota of Kit^wsh/wsh^ mice was responsible for the susceptibility to DSS‐induced colitis, we conducted fecal microbiota transplantation (FMT) experiments to analyze the development of colitis in WT mice after transferring filtered fecal material containing the gut microbiota (Figure 2E). Recipient mice were pretreated with ABX to facilitate colonization of the donor microbiota. DSS‐treated WT mice that received microbiota from Kit^wsh/wsh^ mice developed more severe colitis; weight loss, colon length shortening, colon tissue damage, and proinflammatory cytokine expression were aggravated compared with that receiving WT microbiota (Figure 2F–I). Furthermore, fecal microbiota from WT mice caused Kit^wsh/wsh^ mice to be more resistant to colitis, whereas microbiota from Kit^wsh/wsh^ mice induced severer colitis in Kit^wsh/wsh^ mice (Figure 2J–N). Thus, these data suggest that the altered microbiota in Kit^wsh/wsh^ mice is responsible for the increased susceptibility to DSS‐induced colitis.

### 2.4 GI Dysmotility Altered the Gut Microbial Composition

**Figure 3:**
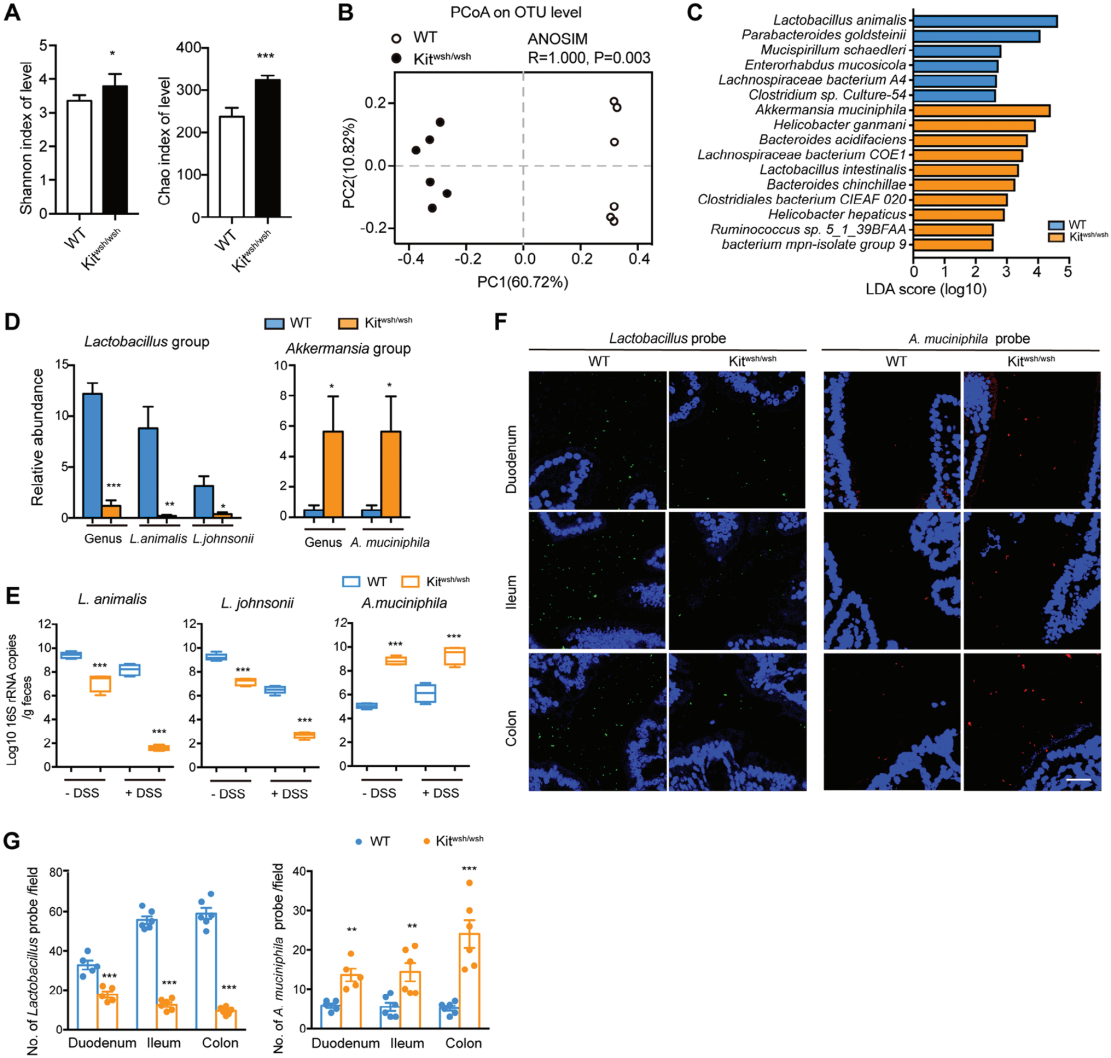
GI dysmotility altered the gut microbiota composition. A–D) 16S rRNA gene sequencing was used to analyze the stool samples from WT (n = 6) and Kit^wsh/wsh^ (n = 6) mice. A) α‐Diversity was characterized by Shannon index and Chao index. B) β‐Diversity analysis of gut microbiota of WT and Kit^wsh/wsh^ mice. Ordination plot based on the PCoA using Bray‐Curtis demonstrates the taxonomic variations of microbial communities across the two groups of mice. C) Differences in microbial taxa at species levels between WT and Kit^wsh/wsh^ mice were calculated by LDA effect size (LEfSe). Mann‐Whitney test was used with a statistical significance cutoff of P < 0.05 and LDA score > 2.5. D) Relative abundances of the genus *Lactobacillus*, *Akkermanisa* and their species detected in 16S rRNA sequencing in fecal samples of WT and Kit^wsh/wsh^ mice. E) Absolute abundances of *L. animalis*, *L.johnsonii*, and *A. muciniphila* in DSS treated or non‐treated WT and Kit^wsh/wsh^ mice analyzed by QPCR. F) Representative fluorescent in situ hybridization images of *Lactobacillus* and *A. muciniphila* in duodenum, ileum and colon tissues of WT and Kit^wsh/wsh^ mice. Scale bar, 25 µm. G) Quantification of *Lactobacillus* and *A. muciniphila* positive probes per field of F. In A, D, E and G, data represent mean ± SEM; *P < 0.05, **P < 0.01, ***P < 0.001 by two‐sided Student *t* test.

To determine the difference in microbial composition between the WT and Kit^wsh/wsh^ mice, we used 16S ribosomal RNA (rRNA) gene sequencing to analyze fecal material from the two groups of mice. Compared to WT samples, Kit^wsh/wsh^ fecal samples displayed increased species richness, as assessed by α‐diversity (**Figure** **3**A). Multidimensional principal coordinates analysis (PCoA) at the operational taxonomic unit (OTU) level based on Bray‐Curtis dissimilarity index revealed significant compositional differences between WT and Kit^wsh/wsh^ samples (ANOSIM, R = 1.000, P = 0.003) (Figure 3B). Linear discriminant analysis (LDA) effect size (LEfSe) method was used to identify the most differentially abundant genera and species between WT and Kit^wsh/wsh^ samples. The Kit^wsh/wsh^ samples exhibited an enrichment in *Akkermansia muciniphila*, but a relative loss of *Lactobacillus animalis* (Figure 3C). In the *Lactobacillus* genus, the abundance of *Lactobacillus johnsonii* was decreased in Kit^wsh/wsh^ samples, in addition to *L. animalis* (Figure 3D). These alterations in *Lactobacillus* and *Akkermansia* were further confirmed by qPCR (Figure 3E). The difference in *L. animalis* and *L. johnsonii* between WT and Kit^wsh/wsh^ mice was further exaggerated following DSS treatment; however, that of *A. muciniphila* showed no further exaggeration (Figure 3E). In addition, we used fluorescent in situ hybridization to examine the spatial distribution of *Lactobacillus* and *A. muciniphila* along the gut tracts of WT and Kit^wsh/wsh^ mice. The abundance of the two bacterial groups in the colon showed the greatest difference between the WT and Kit^wsh/wsh^ mice (Figure 3F,G).

Next, we investigated how gut motility affects the microbial composition. Slow transit (decreased motility) leads to gradual depletion of easily fermentable substrates, primarily saccharides, due to the prolonged digestion, absorption, and fermentation. Consequently, the limited availability of carbohydrate favors bacteria capable of utilizing alternative energy sources such as dietary or host‐derived proteins.^[^ ^18^ ^]^ This hypothesis may potentially explain the microbial change observed in our study, as we observed a decrease in the abundance of lactobacilli, which are proficient in utilizing carbohydrates,^[^ ^19^ ^]^ whereas an increase in the abundance of *A. muciniphila*, a bacterium capable of utilizing mucin as an energy source.^[^ ^20^ ^]^ Therefore, we studied whether carbohydrate deprivation could alter the microbial composition. WT mice were fed either an isocaloric low‐carbohydrate and high‐protein diet (LCHD, 28.9% carbohydrate, 60.0% protein, and 11.1% fat as percentages of calories) or a control diet (CD, 67.4% carbohydrate, 21.5% protein, and 11.1% fat as percentages of calories) for 4 weeks (Figure S4A, Supporting Information). Throughout the feeding period, food intake remained similar between LCHD and CD‐fed groups (Figure S4B, Supporting Information). Notably, the abundance of *Lactobacillus* decreased and the abundance of *A. muciniphila* increased in fecal samples from LCHD‐fed mice (Figure S4C, Supporting Information). However, the degree of microbial alteration between mice LCHD and CD‐fed mice exhibits a lesser magnitude compared to the disparity observed between Kit^wsh/wsh^ and WT mice (Figure 3D,E compared to Figure S4C, Supporting Information). This could be attributed to two factors: first, the duration of the LCHD feeding period might not have been long enough to induce sufficient alterations; second, other factors besides carbohydrate deprivation resulting from decreased gut motility could also contribute to the modulation of microbial composition. Therefore, these data suggest that the carbohydrate restriction caused by slow transit may partially explain the changes of microbial composition.

c‐Kit has been reported to be expressed on mast cells. To exclude the influence of mast cells on microbiota alteration, we first transferred in vitro‐cultured bone marrow derived mast cells (BMMC) into Kit^wsh/wsh^ mice. Six weeks after reconstitution, the absolute abundance of *L. animalis*, *L. johnsonii*, and *A. muciniphila* showed no significant alteration in Kit^wsh/wsh^ mice reconstituted with BMMC compared to non‐reconstituted Kit^wsh/wsh^ mice (Figure S5A, Supporting Information). Additionally, we used a mouse model of mast cell deficiency, named Cpa3‐cre; Mcl^fl/fl^, to confirm the above results. The absolute abundances of *Lactobacillus* and *A. muciniphila* were comparable between Mcl^fl/fl^ and Cpa3‐cre; Mcl^fl/fl^ mice (Figure S5B, Supporting Information). Besides, it was reported that very low number of mast cells was present in the bowel mucosa of normal C57/BL6 mice.^[^ ^21^ ^]^ Thus, the alteration of the gut microbiota in Kit^wsh/wsh^ mice was attributed to ICC loss and consequent dysmotility and not to mast cells.

### 2.5 Linoleic Acid Accumulated in Mice with GI Dysmotility and Increased the Susceptivity to DSS‐Induced Colitis

**Figure 4:**
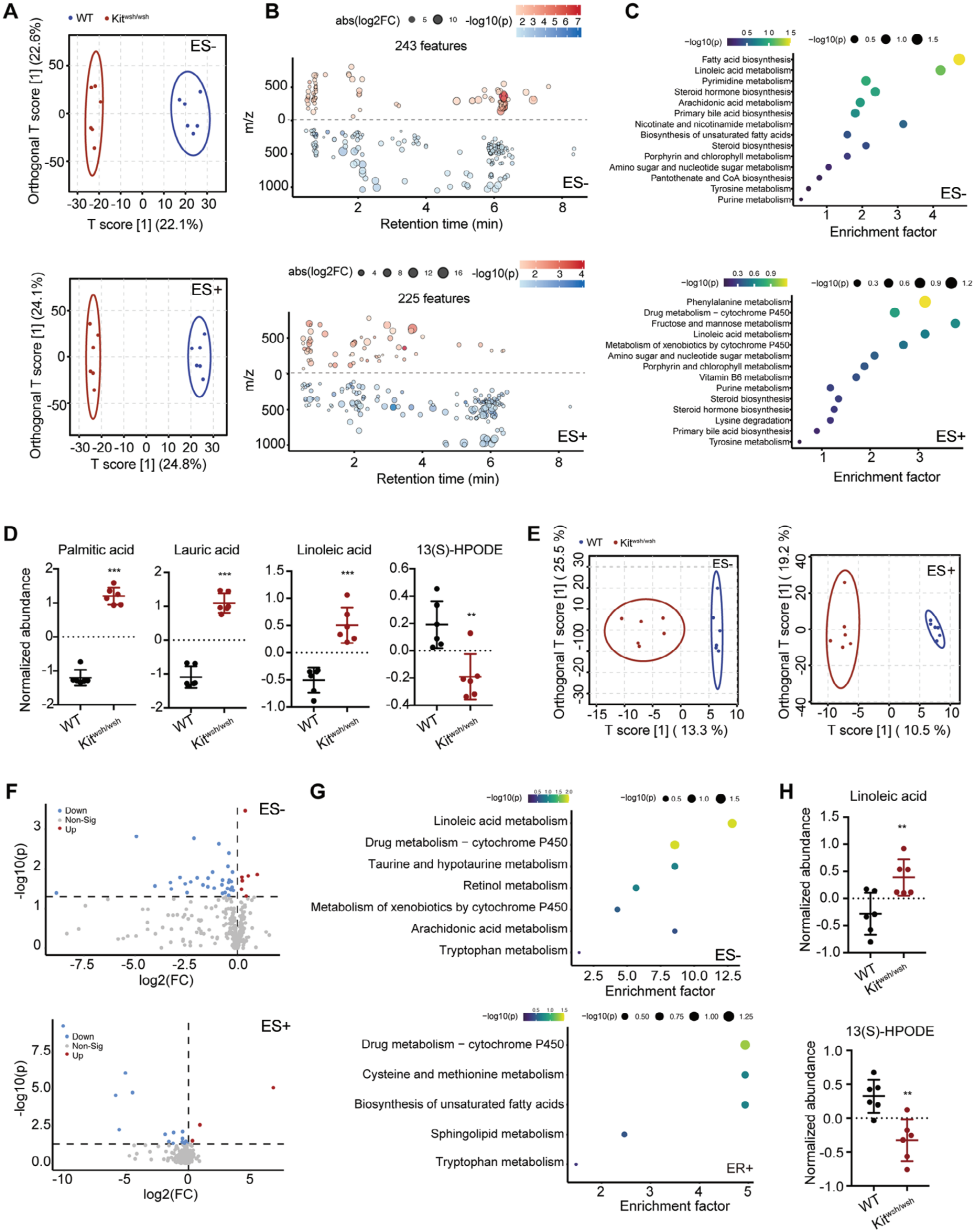
Linoleic acid was enriched in metabolic profiles of both fecal and colonic tissue samples from Kit^wsh/wsh^ mice. Fecal A–D) and colonic tissue E–H) samples from WT (n = 6) and Kit^wsh/wsh^ (n = 6) mice were subjected to untargeted metabolomics liquid chromatography–mass spectrometry analysis. A,E) OPLS‐DA score plots showing all peak features in negative (ES−) and positive (ES+) ion modes. B,F) Scatter plots B) and volcano plots F) of the peak features of metabolites that significantly changed in Kit^wsh/wsh^ mice in ES− mode and ES+ mode. Red and blue circles indicate the significantly increased and decreased metabolites, respectively (fold change >1.5; P < 0.05), in Kit^wsh/wsh^ mice compared with those of WT group. In B, the color tone indicates P: a dark color indicates a small P. The size of the dot indicates the fold change of corresponding peak features. C,G) Dot plots showing the enriched pathways in Kit^wsh/wsh^ mice in ES− mode and ES+ mode. D,H) The normalized abundance of long/medium‐chain fatty acids in WT and Kit^wsh/wsh^ mice. In D and H, data represent mean ± SEM; **P < 0.01, ***P < 0.001 by two‐sided Student *t* test.

Next, untargeted liquid chromatography‐mass spectrometry (LCMS) was applied to explore the metabolic profiling of both fecal and colonic tissue samples from WT and Kit^wsh/wsh^ mice. With fecal samples, a total of 11 353 and 9222 peak features were identified in positive (ES+) and negative (ES−) ion modes, respectively. These peaks were further clustered by orthogonal partial least squares discriminant analysis (OP‐LSDA). OPLS‐DA plots demonstrated a clear separation between the metabolic profile of the WT and Kit^wsh/wsh^ groups in both ES+ and ES‐ modes (**Figure** **4**A). There were 243 and 225 peak features with significant changes in peak intensity in ES‐ (84 upregulated and 159 and downregulated peaks, respectively) and ES+ (61 and 164 upregulated and downregulated peaks, respectively) modes, respectively (Figure 4B). Further, we identified the metabolic pathways significantly altered in Kit^twsh/wsh^ mice. The top enriched pathway included fatty acid biosynthesis, linoleic acid metabolism, pyrimidine metabolism in ER‐ mode and phenylalanine metabolism, drug metabolism – cytochrome P450, fructose and mannose metabolism in ER+ mode (Figure 4C). As fatty acid biosynthesis and linoleic acid metabolism showed the most significant enrichment in Kit^wsh/wsh^ mice, we examined the altered metabolites in these two pathways. Among these metabolites, the abundance of long chain fatty acids (LCFA) including palmitic acid, lauric acid, and linoleic acid were significantly increased in Kit^wsh/wsh^ group, while 13(S)‐HPODE decreased (Figure 4D).

With colonic tissue samples, we identified 603 and 455 peak features in positive and negative ion modes, respectively. OPLS‐DA analysis showed distinct separation between the metabolic profiles of the two groups (Figure 4E). We found 40 upregulated and 27 downregulated peaks in ES‐ mode, and 5 upregulated and 22 downregulated peaks in ES+ mode (Figure 4F). The enriched pathways in Kit^wsh/wsh^ mice included linoleic acid metabolism, drug metabolism‐cytochrome P450, and taurine and hypotaurine metabolism in ES‐ mode, and drug metabolism‐cytochrome P450, cysteine and methionine metabolism, and biosynthesis of unsaturated fatty acids in ES+ mode (Figure 4G). Consistent with our observations in fecal samples, linoleic acid metabolism pathway was significantly enriched in Kit^wsh/wsh^ mice. Significant changes were observed in two metabolites within this pathway: an increase in linoleic acid level and a decrease in 13(S)‐HPODE level in Kit^wsh/wsh^ colonic tissue (Figure 4H).

**Figure 5:**
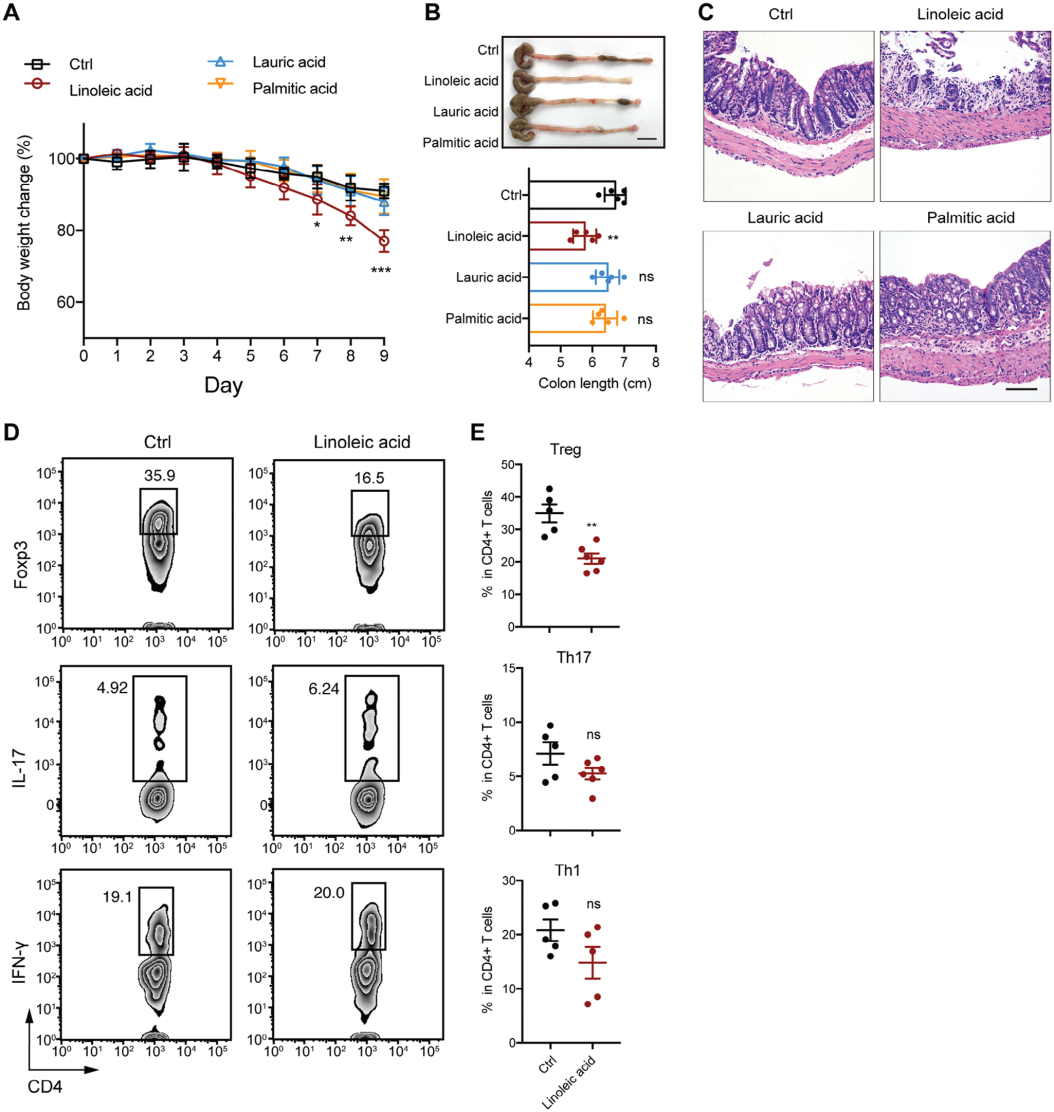
Linoleic acid treatment increased the susceptivity to DSS‐induced colitis by modulating the abundance of Treg. WT mice (n = 5 for each group) were gavaged with 1 g kg^−1^ body weight linoleic acid, lauric acid and palmitic acid respectively every day from 14 days before DSS treatment. The body weight changes A), colon lengths B), representative images of H&E‐stained colon sections C), and colonic infiltration of Treg, Th17, Th1 D and E) in each animal group were monitored or analyzed. Scale bar in C, 100 µm. In A, B, E, data represent mean ± SEM; *P < 0.05, **P < 0.01, ***P < 0.001, ns, not significant by two‐sided Student *t* test E) and one‐way ANOVA with Dunnett's post hoc test A and B).

To investigate whether these altered fatty acids (Figure 4D,H) contributed to the increased susceptivity to DSS of Kit^wsh/wsh^ mice, we gavaged WT mice with palmitic acid, lauric acid, and linoleic acid before DSS treatment, respectively. Only mice gavaged with linoleic acid showed a significant decrease of body weight, shortened colon length and increased morphological damage (**Figure** **5**A–C). Given the infiltration of Th17 and Treg altered in DSS treated Kit^wsh/wsh^ mice (Figure S2, Supporting Information), we speculated that linoleic acid might increase the susceptibility to DSS through modulation of intestinal immune cells. Thus, we next examined the effect of linoleic acid gavage on the infiltration of Th1, Th17, and Treg in the cLP from mice with DSS treatment. The infiltration of Treg significantly decreased in mice gavaged with linoleic acid (Figure 5D,E), which is in consistent with that in Kit^wsh/wsh^ mice (Figure S2A, Supporting Information). However, the infiltration of Th1 and Th17 was similar between mice gavaged with vehicle control and linoleic acid (Figure 5D,E). Together, these data suggest that the enrichment of linoleic acid contributed to the increased susceptibility to DSS, partly through reducing Treg infiltration.

### 2.6 Linoleic Acid Inhibited Treg Cell Differentiation in a Macrophage‐ Dependent way

**Figure 6:**
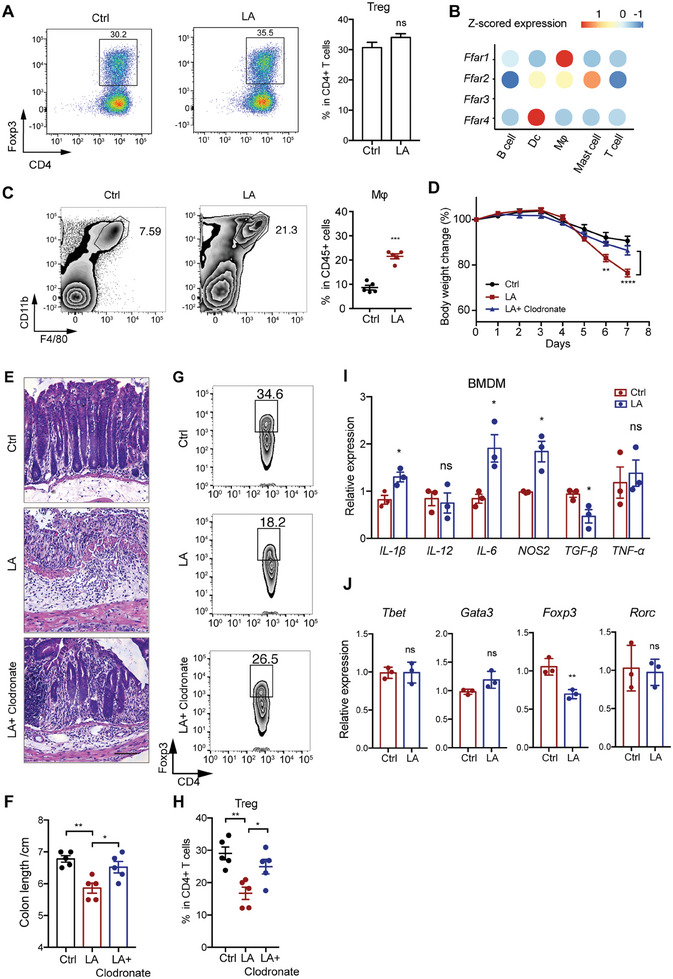
Linoleic acid inhibited Treg cell differentiation through inducing macrophage infiltration and proinflammatory cytokine expression. A) Naïve CD4^+^ T cells which were isolated and purified from murine spleen and treated with vehicle control or 0.05 mM linoleic acid (LA) for 3 days under the condition of 2 ng mL^−1^ TGF‐β and 5 ng mL^−1^ IL‐2. B) Dot plot of average Z‐scored expression of fatty acid receptors in intestinal immune cell subsets from the mouse single cell sequencing database Mouse Cell Altas. The color represents the Z‐scored expression of fatty acid receptors. Mφ, macrophage. Dc, dendritic cell. C) Flow cytometry analysis of macrophage infiltration in cLP from DSS treated mice with Ctrl or LA gavage. D–H) WT mice (n = 6 for each group) were gavaged with or without 1 g kg^−1^ body weight LA every day from 14 days before DSS treatment. To deplete macrophages, clodronate was administrated 3 days before and every 2 days during DSS treatment. The body weight changes D), representative images of H&E‐stained colon sections E), colon lengths F), and infiltration of Treg G and H) in each group were monitored or analyzed. I) QPCR analysis of cytokine expression in bone marrow derived macrophages (BMDMs) treated with or without LA for 3 days. J) QPCR analysis of naïve CD4^+^ T cells treated with conditioned media from BMDMs in I. Scale bar (E), 50 µm. In A, C, D, F, H‐J, data represent mean ± SEM; *P < 0.05, **P < 0.01, ***P < 0.001, ns, not significant by two‐sided Student *t* test (A, C, I and J), one‐way ANOVA with Dunnett's post hoc test (D, F and H).

We next investigated the mechanism of linoleic acid (LA) regulating Treg infiltration. LA treatment in vitro could not inhibit Treg cell differentiation (**Figure** **6**A), indicating LA did not impact directly on CD4^+^ T cells and other type of cells might mediate this effect. To figure out which type of the cell, we first investigated the expression of free fatty acid receptors (FFAR) including FFAR1, FFAR2, FFAR3, and FFAR4 on murine intestinal immune cells using a murine single cell database Mouse Cell Atlas.^[^ ^22^ ^]^ Among the four receptors, FFAR1 and FFAR4 are the receptors for LCFA. FFAR1, which receptor LA showed a higher affinity for,^[^ ^23^ ^]^ showed the highest expression on macrophages (Figure 6B). The infiltration of macrophage was also increased in cLP of DSS treated‐ mice gavaged with LA (Figure 6C). To investigate whether the decrease in Treg cells induced by LA was mediated by macrophages, we used clodronate to deplete macrophages prior to DSS treatment. Macrophage depletion decreased the susceptivity to DSS‐induced colitis in mice gavaged with LA, including mitigated body weight loss, morphological damage, and colon length shortening (Figure 6D–F). Macrophage depletion also led to the restoration of Treg cell populations in mice gavaged with LA (Figure 6G,H). Next, we treated bone marrow derived macrophages (BMDM) with LA and collected the conditioned medium form these cells to treat naïve CD4^+^ T cells. LA treatment upregulated *Il‐1β*, *Il‐6* expression, while downregulated *Tgf‐β* expression in BMDMs (Figure 6I). The conditioned medium from LA‐treated BMDM inhibited *Foxp3* expression in naïve CD4^+^ T cells (Figure 6J). Together, these data suggest that LA could increase macrophage infiltration and induce proinflammatory cytokines expression in macrophage, which inhibited Foxp3 expression and subsequent Treg cell differentiation and resulted in increased susceptivity to DSS‐induced colitis.

### 2.7 Significantly Altered Metabolites in Phenylalanine Metabolism had no Impact on Colitis Susceptibility

To explore the potential contribution of phenylalanine metabolism, top enriched in ER+ mode of fecal samples from Kit^wsh/wsh^ mice, to the increased susceptivity of Kit^wsh/wsh^ mice to DSS‐induced colitis, we first identified the metabolites within this pathway that displayed significant alterations. The abundance of phenylacetylglycine, phenylacetaldehyde, and L‐tyrosine increased significantly in Kit^wsh/wsh^ mice (Figure S6A, Supporting Information). We next treated the WT mice with each of these metabolites at the concentration of 200 mg kg^−1^ body weight per day individually for 2 weeks,^[^ ^24^ ^]^ followed by induction of colitis using DSS. Mice receiving the respective metabolite treatment displayed similar changes in body weight, colon length, colonic morphology, and inflammatory cytokine expression with control mice (Figure S6B–E, Supporting Information). Additionally, the ratio of Treg and Th17 in colonic tissue was comparable among all four group (Figure S6F,G, Supporting Information). These data indicate that the phenylalanine metabolism pathway does not exert an effect on susceptibility to colitis.

### 2.8 *L. Animalis* reduced Susceptivity to Colitis through Converting LA

As Kit^wsh/wsh^ mice displayed an altered microbial alteration, we next investigate the relationship between linoleic metabolism and the altered abundance of *Lactobacillus* and *A. muciniphila*. We initially performed redundancy analysis (RDA) using bacteria species that different between Kit^wsh/wsh^ and control mice, and the altered metabolites in fatty acid biosynthesis and linoleic acid metabolism pathway including palmitic acid, lauric acid, linoleic acid, and 13(S)‐HPODE. RDA analysis revealed a significant negative relationship between *L. animalis* and linoleic acid (Figure S7A, Supporting Information), which was further confirmed by Pearson correlation analysis (Figure S7B, Supporting Information).

Certain bacterial species including *Enterococcus*, *Lactobacillus*, *Bifidobacterium*, *Ruminococcus*, and *Lachnospiraceae*, can convert LA into conjugated linoleic acid (CLA) through the catalytic activity of linoleic acid isomerases (LAIs). CLA has been demonstrated to possess not only non‐harmful but also beneficial effects on heath.^[^ ^25^ ^]^ We first used the protein sequence of *Lactobacillus plantarum* LAI as a query in a BLAST to search the *L. animalis* genome, and discovered the presence of a homologous enzyme (Figure S7C, Supporting Information). To investigate the ability of *L. animalis* to reduce LA level in vivo, WT mice were initially subjected to antibiotic treatment and LA gavage at the same time for one month to eliminate the original *L. animalis* and to enrich LA levels, followed by a 2‐week gavage of *L. animalis*. Fecal samples were collected before and after *L. animalis* administration (Figure S7D, Supporting Information). The administration of *L. animalis* significantly decreased the levels of LA (Figure S7E,F, Supporting Information).

**Figure 7:**
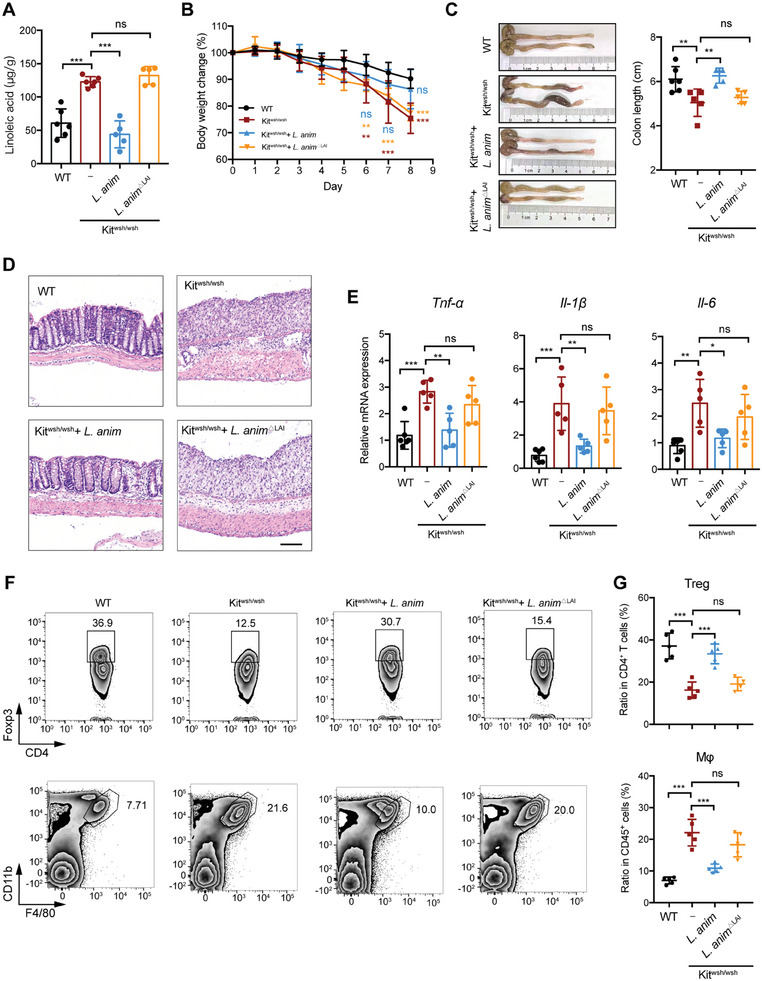
*L. animalis* mitigated the susceptivity to colitis, which is dependent on its ability to convert LA. Kit^wsh/wsh^ mice (n = 5/group) were gavaged with PBS, *L. animali* (*L. anim*) or *L. anim* ^△LAI^ for 4 weeks, followed by a 2.5% DSS treatment for 7 days and water 1 day. WT mice (n = 6) served as control. A) GC–MS quantification of the amount of LA in feces from WT, Kit^wsh/wsh^, Kit^wsh/wsh^ receiving *L. anim*, and Kit^wsh/wsh^ receiving *L. anim* ^△LAI^ mice. B–G) The body weight changes B), colon lengths C), representative images of H&E‐stained colon sections D), proinflammatory cytokine expression E) and infiltration of Treg and Mφ in colonic tissue F and G) were monitored or analyzed. Scale bar (D), 100 µm. In A‐C, E, and G, data represent mean ± SD; *P < 0.05, **P < 0.01, ***P < 0.001, ns, not significant by one‐way ANOVA with Dunnett's post hoc test.

Given the ability of *L. animalis* to convert LA, our subsequent investigation aimed to determine whether restoration of *L. animalis* could mitigate the susceptibility to colitis in Kit^wsh/wsh^ mice. We first generated a strain of LAI ‐deficient *L. animalis* (*L. animalis* ^△LAI^) bacteria by replacing the LAI gene with a chloramphenicol resistance gene. A 4‐week administration of *L. animalis* significantly reduced the levels of fecal LA in Kit^wsh/wsh^ mice, whereas the administration of *L. animalis* ^△LAI^ showed no significant changes (**Figure** **7**A). The administration of *L. animalis* resulted in decreased susceptivity to colitis, as evidenced by reductions in body weight loss and colon length shortening, improved colonic morphology, and decreased expression of proinflammatory cytokines (Figure 7B–E). Furthermore, the administration of *L. animalis* led to a decrease in the ratio of macrophage and an increase in the ratio of Treg (Figure 7F,G). However, after losing the ability to convert LA, the *L. animalis* bacteria were unable to alleviate colitis in Kit^wsh/wsh^ mice (Figure 7B–G). In summary, the diminished presence of *L. animalis* in Kit^wsh/wsh^ resulted in a limited covert of LA, leading to the accumulation of LA in the gut and an increased susceptivity to DSS.

### 2.9 Altered Microbial Composition in Patients with CC Contributed to an Increased Susceptibility to Colitis

**Figure 8:**
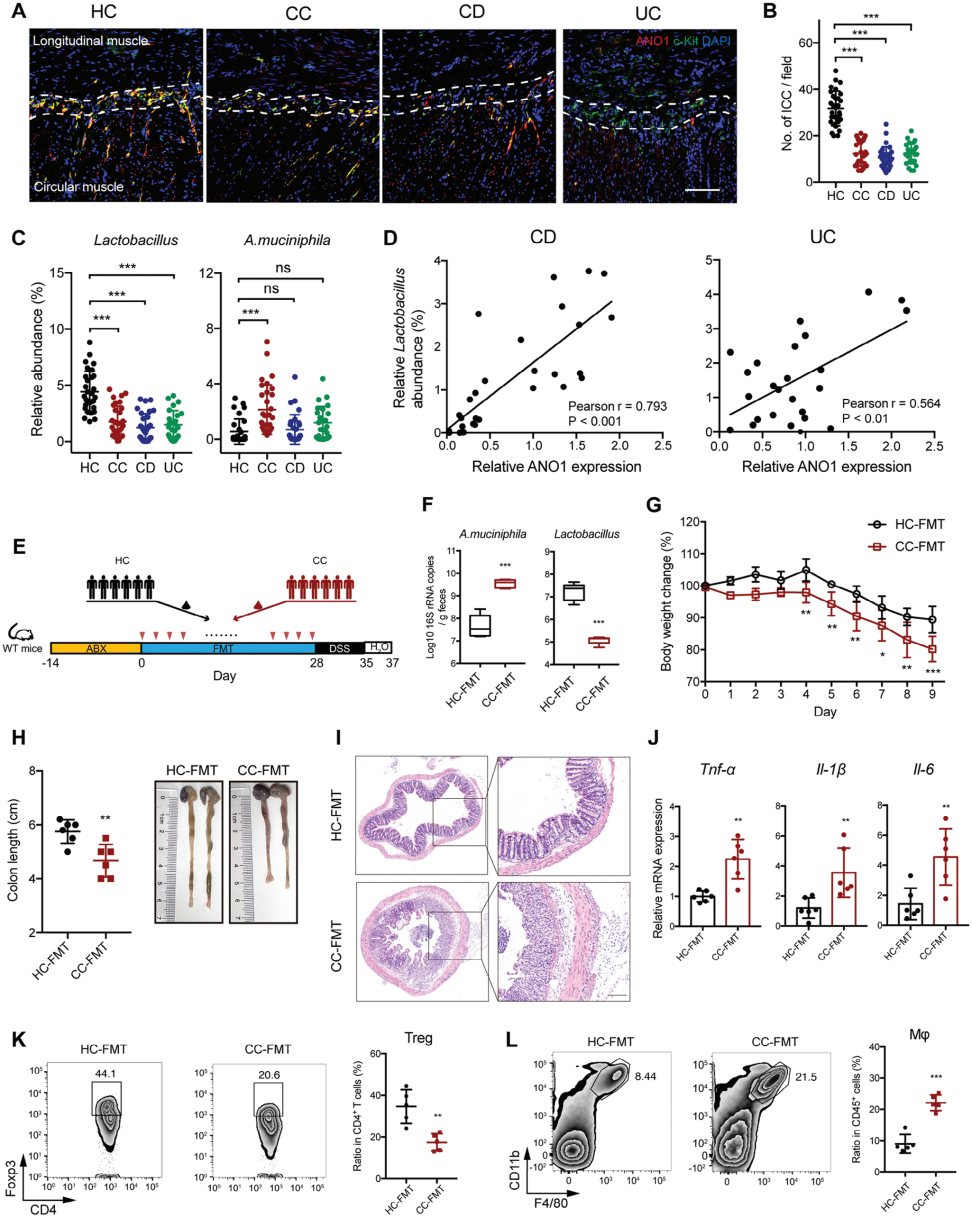
Fecal microbiota was altered in patients with CC, which increased susceptibility to colitis. A) Representative images of interstitial cells of Cajal (ICC) in myenteric plexus (located between the longitudinal muscle and circular muscle) stained using anti‐Anoctamin‐1 (ANO1) and anti‐c‐Kit antibody in colon tissues from CC (n = 30), inflamed region from CD patients (n = 32) and UC patients (n = 25), and normal adjacent tissue from CRC patients (n = 30). Scale bar, 100 µm. B) The number of ICCs per field in A. C) Relative abundance of *Lactobacillus* and *A. muciniphila* in stool samples from healthy donors (HC), CC, CD and UC patients. D) The correlation between the relative abundance of *Lactobacillus* with the expression of ANO1 in inflamed colon tissue from CD and UC patients. E) Experimental scheme of fecal microbiota transfer (FMT). WT mice (n = 6/group) were treated with a cocktail of antibiotics (ABX) for 14 days, and then gavaged with fecal microbiota pooled from 6 healthy controls (HC‐FMT) or 6 patients with chronic constipation (CC‐FMT) every other day for 4 weeks, followed by a 2.5% DSS treatment for 7 days and water 2 days. F) QPCR analysis of the abundance of *A. muciniphila* and *Lactobacillus* in fecal sample from mice receiving FMT. G–L) The body weight changes G), colon lengths H), representative images of H&E‐stained colon sections I), proinflammatory cytokine expression J) and infiltration of Treg K) and macrophage (Mφ) L) in colonic tissue were monitored or analyzed. Scale bar I), 100 µm. In B, C, F‐H, and J‐L, data represent means ± SD; *P < 0.05, **P < 0.01, ***P < 0.001 by one‐way ANOVA with Dunnett's post hoc test (B and C) and two‐sided Student *t* test (F‐H, and J‐L).

Finally, we investigated the number of ICC and the abundance of *Lactobacillus* and *A. muciniphila* in patients with CC, CD, and UC. The number of ICC decreased significantly in colon tissues from CC, and inflamed colon tissues from CD and UC patients compared to that in normal tissues (**Figure** **8**A,B). The relative abundance of *Lactobacillus* was significantly decreased in CC, CD and UC patients (Figure 8C); and showed a positive correlation with ANO1 expression in CD and UC patients, which reflects the number of ICC (Figure 8D). The relative abundance of *A. muciniphila* was increased in patients with CC, however similar between healthy controls and patients with CD or UC (Figure 8D), possibly owing to the disrupted mucus layer, which is the carbon and nitrogen source for *A. muciniphila*, in those patients (Figure S8, Supporting Information).

To directly investigate the contribution of altered microbiota in patients with CC to colitis progression, we performed an FMT experiment. WT mice underwent a 2‐week treatment with an antibiotic cocktail to eliminate the original bacteria. The mice were then administered with fecal microbiota from a pool of samples derived from either 6 healthy controls or 6 patients with CC, once every two days, for a duration of 4 weeks (Figure 8E). Analysis of the fecal samples from mice receiving fecal microbiota from CC patients revealed a reduction in the abundance of *Lactobacillus*, and an increase in the abundance of *A. muciniphila* (Figure 8F). This indicates the successful transfer of human‐derived fecal microbiota to the mice. Mice that received fecal microbiota from CC patients exhibited increased susceptibility to DSS‐induced colitis, characterized by higher body weight loss, shortened colon length, increased mucosal damage, and elevated expression of proinflammatory cytokines (Figure 8G–J). Furthermore, these mice demonstrated a decreased ratio of Treg and an increased ratio of macrophages (Figure 8K,L). Taken together, these data suggest that the altered microbiota in CC patients may contribute to an increased susceptibility to colitis.

## 3 Discussion

Disrupted GI motility is highly prevalent in patients with IBD, but whether it is a cause of the disease remains unknown. In this study, we demonstrated that decreased GI motility predisposed the host to colitis development through altering the abundance of gut *Lactobacillus* (*L. animalis* and *L. johnsonii* in the majority) and *A. muciniphila*. The decreased abundance of *L. animalis* led to accumulation of LA, which resulted in inhibited Treg cell differentiation, and subsequent exacerbated mucosal inflammation. Thus, our study provided novel insights into a vital role for GI motility in maintaining gut homeostasis by the regulation of gut microbiota and LCFA metabolism.

The relationship between GI motility and the gut microbiota is complex and has not yet been well elucidated. Although GI motility and the gut microbiota can influence each other, recent evidence supports the idea that GI motility is the driving force behind the composition and metabolism of the gut microbiota.^[^ ^26^ ^]^ The flow and peristaltic mixing of the gut plays a fundamental role in the rate of bacterial growth. Different bacteria have different growth rates; thus, different bacterial species dominate under different types of gut flow.^[^ ^27^ ^]^ The growth rate of *A. muciniphila* was much lower than that of *Lactobacillus*; under the condition of longer colonic transit time (decreased GI motility), the abundance of species with lower growth rates, such as *A. muciniphila*, might increase gradually, while that of species with higher growth rates, such as *Lactobacillus*, might decrease owing to limited nutrition. Thus, it seems that GI motility acts as the selection factor that chooses bacterial groups with the most suitable growth rate for survival.

Decreased GI motility led to decreased abundance of *Lactobacillus* and increased abundance of *A. muciniphila*. The decrease in *Lactobacillus* abundance has a close relationship with the pathogenesis of colitis, experimental autoimmune encephalomyelitis, and other immune‐related disease.^[^ ^28^ ^]^ A meta‐analysis of 27 randomized controlled trials demonstrated that combination treatment with *Lactobacillus* and other probiotics confers a significant advantage in UC and may have a significant effect on CD in overall.^[^ ^29^ ^]^ The protective effect of *Lactobacillus* might be attributed to their inhibiting impact on intestinal inflammation, and for example *L. johnsonii* was shown to activate gut CD206^+^ macrophages and increase IL‐10 expression, which alleviated colitis in mice^[^ ^30^ ^]^ recently. The relationship between *A. muciniphila* and colitis pathogenesis has been widely investigated recently. A high‐fat diet promoted colitis pathogenesis in DSS‐treated WT mice and *Il10* ^−/−^ mice, the mechanism of which can be attributed to the increased abundance of the mucus‐degrading bacteria *A. muciniphila* and *Bacteroides fragilis* in the gut.^[^ ^31^ ^]^ The increased abundance of *A. muciniphila* could lead to increased IL‐1α release and increased susceptibility to DSS‐induced colitis in *Il33* ^−/−^ mice.^[^ ^32^ ^]^ Consistently, *A. muciniphila* has been demonstrated to have the highest significant decrease in abundance in Tak1^△M/△M^ mice, which showed complete resistance to chemical‐induced colitis and CRC.^[^ ^33^ ^]^ *A. muciniphila* was also correlated with exacerbated tumorigenesis in mice that received fecal microbiota from CRC patients.^[^ ^34^ ^]^ The promoting role of *A. muciniphila* in colitis /CRC pathogenesis might be attributed to its degradation of mucin,^[^ ^20^ ^]^ which is the main component of the mucus layer. The degradation has been shown to result in the erosion of the mucus layer and correlates closely with intestinal inflammation and the colonization of intestinal pathogens.^[^ ^35^ ^]^ However, administration of pasteurized *A. muciniphila* has been reported to improve DSS‐induced colitis and blunt the pathogenesis of colitis‐associated colorectal cancer.^[^ ^36^ ^]^ This contradictory effect of *A. muciniphila* on colitis and CRC pathogenesis might be due to whether the bacteria used when treating animals is live or not.

A high dietary intake of LA was reported to be associated with increased risk of disease development and flare in UC,^[^ ^37^ ^]^ however the underlying mechanism is not well studied. We revealed that LA exerted proinflammatory effect on macrophages, which increased the expression of *IL‐6*, and *IL‐1β* and decreased the expression of *TGF‐β*, inhibited Treg cell differentiation, and increased susceptivity to DSS‐induced colitis. Besides, several other aspects might also explain the positive relation between LA uptake and colitis pathogenesis. Linoleic acid induced T cell proliferation at a low concentration.^[^ ^38^ ^]^ In human, LA is the major dietary source to synthesize arachidonic acid, which has been reported to entail a risk for developing IBD^[^ ^39^ ^]^ and accumulate in the inflamed mucosa of IBD patients.^[^ ^40^ ^]^

Overall, our study found that defects in GI motility could lead to an increased risk of colitis pathogenesis via modulation of the composition of the gut microbiota and LA metabolism. This finding suggests that people with motility disorders, such as constipation, might have a potential risk of colitis development, thus the specific modulation of microbiota and LCFA signaling can be promising to protect them from intestinal inflammation.

## 4 Experimental Section

### Patients

All patients with CC (n = 30), CD (n = 32), UC (n = 25) or colorectal cancer (n = 30) were recruited from the Department of Gastroenterology, the Shanghai Tenth People's Hospital of Tongji University (Shanghai, China) from 2018 to 2020. Full‐thickness colonic samples from CC patients, inflamed region of CD and UC patients who underwent a surgery were collected. Normal control samples were taken at least 10 cm away from any macroscopically visible lesion from patients with colorectal cancer without previous history of abdominal surgery, inflammatory bowel disease or intestinal obstruction. The fecal samples from patients with CC, CD and UC were also collected. Fecal samples healthy volunteers upon routine physical examination in Shanghai Tenth People's Hospital of Tongji University served as normal controls. Individuals who received antibiotics or probiotics within latest 8 weeks were excluded. The protocols for patient sample collection were approved by the Institutional Review Board for Clinical Research of the Shanghai Tenth People's Hospital of Tongji University (permit 2018‐KN22‐01). Written informed consent was also obtained from all participants before the study protocol began. The study was conducted in accordance with the Declaration of Helsinki and Rules of Good Clinical Practice. The baseline characteristics are described in Table S1 (Supporting Information).

### Animals

WT C57BL/6J mice were from Shanghai Model Oragnisms Center (Shanghai, China). Kit^wsh/wsh^ C57BL/6J mice were from Jackson Laboratory (BarHarbor, ME). All mice were housed in a specific pathogen‐free environment in the Animal Laboratory Unit, Tongji University, China. Age‐ and sex‐matched mice and littermate controls were used at 8–10 weeks of age, and were randomly assigned into different treatment groups. All animal studies were approved by the Institutional Animal Care and Use Committee of Tongji University (permit SHDSYY‐2018‐4157).

### Microorganisms

*L. animalis* (CGMCC 1.2623, same with ATCC 35 046), *L. johnsonii* (CGMCC 1.3348, same with ATCC 33 200) were obtained from the China General Microbiological Culture Collection Center (Beijing, China). *A. muciniphila* (ATCC BAA‐835) was purchased from ATCC. Lactobacillus was cultured under aerobic conditions on Lacobacilli MRS Broth and the colonies were formed on MRC Agar. *A. muciniphila* was cultured under anaerobic conditions on in brain heart infusion (BHI) + 2.5 g L^−1^ Type‐III hog gastric mucin. All microorganisms were cultured at 37 °C. All culture mediums were purchased from BD (MD, USA).

### Tissue RNA Extraction and QPCR

Total RNA of human or mouse tissues were isolated using Trizol reagent (Invitrogen, 15 596 026) according to the manufacturer's instructions, and then reverse transcribed using PrimeScript RT Master Mix (Takara, RR036A). Quantitative PCR (QPCR) was performed with SYBR Green Real‐time PCR Master Mix (Takara, RR820A) and quantified by the QuantStudio 6 Flex Real‐Time PCR System (Applied Biosystems). The sequences of primers are listed in Table S2 (Supporting Information).

### Immunofluorescence

For immunofluorescence staining analysis, frozen sections were blocked with 10% goat serum or donkey serum, then incubated with primary antibody overnight at 4 °C. These antibodies included fluorochrome‐conjugated anti‐human CD4 (clone RPA‐T4, Tonbo Biosciences, 50‐0049), anti‐human CD8 (clone RPA‐T8, BD Biosciences, 555 366), anti‐human CD68 (clone eBioY1/82A, ebioscience, 11‐0689‐41); and non‐fluorochrome‐conjugated anti‐CD117/c‐Kit (R&D Systems, AF1356), anti‐mucin‐2 (Santa Cruz Biotechnology, sc‐59859), anti‐TMEM16A/Anoctamin 1 (Abcam, ab53212), anti‐Claudin‐1 (Abcam, ab211737), anti‐JAM‐A (Abcam, ab180821), and anti‐ZO‐1 (Abcam, ab221547) antibodies. For sections that were incubated with primary antibodies without the fluorochrome conjugation, secondary antibodies were applied for 60 min at room temperature using Alexa Fluor 488 conjugated donkey anti‐goat IgG (Invitrogen, A‐11055) or Alexa Fluor 555 conjugated goat anti‐rabbit IgG (Invitrogen, A27039). Nuclei was counterstained with 4,6‐diamidino‐2‐phenylindole (DAPI).

Images were acquired using a Leica TCS SP8 confocal microscope. For cell quantification, 5 random fields of the specimen were photographed (20 x magnification), then the cells of interest in each field is counted and the average of cell number per field was calculated for each slide. All slides were counted separately by two individuals blinded to the clinical or treatment group.

### Gastrointestinal Transit Time Measurement

Mice were gavaged with 300 µl of 6% carmine red (Sigma–Aldrich, C1022) suspended in 0.5% methylcellulose (Sigma–Aldrich, M0512) solution. Following administration of the solution, the mice were allowed free access to food and water ad libitum. Gastrointestinal transit time was measured as the time interval between the begin of gavage and the appearance of the first red fecal pellet.

### DSS‐Induced Acute Colitis

Acute colitis was induced by 2.5% (w/v) DSS with molecular mass of 36–50 kDa (MP Biomedicals) in drinking water for a total of 7 days, followed by a recovery period with regulatory drinking water of 2–3 days. Body weight changes and disease activity index were monitored daily for each mouse. The disease activity index (DAI) ranging from 0 to 4 was assessed as the combined scores of weight loss, stool consistency, and fecal blood as described previously.^[^ ^41^ ^]^ On day 9, mice were killed, and colon tissue were collected for further analysis. To investigate the effect of loperamide‐induced constipation on colitis, WT mice were gavaged with 10 mg kg^−1^ body weight (b.w.) loperamide (Sigma‐Aldrich, L4762) every day from 7 days before DSS treatment to sacrifice. To investigate the effect of *Lactobacillus* on colitis, Kit^wsh/wsh^ mice were treated with an oral gavage of a combination of *L. animalis* and *L. johnsonii* (3:1 ratio, 10^9^ CFU mouse^−1^) from 14 days before DSS treatment to sacrifice. To investigate the effect of LCFAs on colitis, WT mice were treated with an oral gavage of 1 g kg^−1^ b.w. palmitic acid, lauric acid, or linoleic acid respectively every other day from 14 days before DSS treatment to sacrifice. To investigate the effect of phenylalanine metabolism on colitis, WT mice were treated with water as a control or phenylacetylglycine (MedChemExpress, HY‐W015061), phenylacetaldehyde (Sigma–Aldrich, W287407), L‐tyrosine (Sigma–Aldrich, 1 083 710 025) resuspended in drinking water individually at the concentration of 200 mg kg^−1^ per day (based on the assumption of a mouse consuming 5 ml of water daily) for 14 days before DSS treatment.^[^ ^24b^ ^]^

### Histopathology

For morphological analysis, distal colon tissues were fixed with 4%PFA, embedded in paraffin, sectioned with 4‐µm thickness, and followed by hematoxylin and eosin staining. Histological images were captured using Olympus BX51 (Olympus Corp., Tokyo, Japan). Histology was scored as follows:^[^ ^42^ ^]^ inflammatory cell infiltration (score 0–3) 0, The presence of occasional inflammatory cells in the lamina propria; 1, increased numbers of inflammatory cells in the lamina propria; 2, confluence of inflammatory cells, extending into the submucosa; 3, transmural extension of the infiltrate); tissue damage (score 0–3) 0, no mucosal damage; 1, discrete lymphoepithelial lesions; 2, surface mucosal erosion or focal ulceration; 3, extensive mucosal damage and extension into deeper structures of the bowel wall. The combined histological score ranged from 0 (no changes) to 6 (extensive cell infiltration and tissue damage). Histological score was assessed by an independent pathologist who was blinded to the treatment groups.

### Fecal Microbiota Transplantation

For murine samples, fresh fecal pellets were collected directly from sex‐ and age‐matched donor mice, then pooled, mixed with sterile PBS (0.1 g feces in 1 ml PBS), and homogenized immediately. For human samples, feces from 6 patients with CC and 6 healthy controls were pooled respectively. The homogenate was filtered through 100 µm strainer. For fecal microbiota transplant (FMT), recipient mice were pre‐treated with a cocktail of antibiotics (ABX) including ampicillin (1 mg mL^−1^), streptomycin (5 mg mL^−1^), colistin (1 mg mL^−1^, all from Wako Chemicals) in drinking water for 2 weeks to deplete distinct taxa of bacteria. Recipient mice were administrated with the above filtered donor fecal homogenate by oral gavage (300 µl per mouse) one day after ABX treatment every other day over a period of 10 days.

### 16S rRNA Sequencing and Analyses

Total fecal DNA was extracted using the QIAamp DNA Stool Mini Kit (Qiagen, Hilden, Germany) according to the manufacturer's protocols. The V3‐V4 region of 16S rRNA gene was amplified with barcode‐indexed primers 338F (5′‐ACTCCTACGGGAGGCAGCA‐3′) and 806R (5′‐GGACTACHVGGGTWTCTAAT‐3′), using TransStart FastPfu Polymerase. Amplicons were then purified by gel extraction (AxyPrep DNA GelExtraction Kit, Axygen Biosciences, Union City, CA, USA) and were quantified using QuantiFluor‐ST (Promega, USA). The sequencing reaction was conducted using Illumina MiSeq instrument (Illumina, San Diego, USA). The data were analyzed by Majorbio Bio‐pharm Biotechnology (www.i‐sanger.com).

### Fecal Bacteria DNA Extraction and QPCR

Freshly collected mouse or human fecal samples were immediately frozen and stored in −80 °C. Total DNA was extracted using the QIAamp DNA Stool Mini Kit (Qiagen, Hilden, Germany) according to the manufacturer's protocols. QPCR was performed with 100 ng fecal DNA as template and SYBR Green Real‐time PCR Master Mix (Takara, RR820A). QPCR was done with Step One Real‐Time PCR System (Applied Biosystems). The sequences of primers are listed in Table S2 (Supporting Information). The standard curve for absolute quantification was generated by serial dilution of pcDNA3.1 plasmid in which 16S rRNA target sequence from each species was cloned.

### Fluorescence in Situ Hybridization (FISH) of Bacteria and Mucin Staining

The colonization of *Lactobacillus* and *A. muciniphila* in colonic mucosal tissue was detected by FISH analysis using 16S rRNA probe tagged with FAM and Cy3 respectively (BioSune Biotechnology, Shanghai). In situ hybridization of bacteria was performed as previously described.^[^ ^43^ ^]^ Briefly, colon tissues were fixed in Carnoy's fixative (60% methanol, 30% chloroform, 10% acetic acid) and then paraffin‐embedded. The colon sections were de‐paraffinized and incubated with 10 µg mL^−1^ FISH probe diluted in hybridization buffer (0.9 M NaCl, 20 mM Tris‐HCl [pH 7.4], 0.01% sodium dodecyl sulfate, 10% formamide) at 48 °C overnight. Slides were washed with washing buffer (0.9 M NaCl, 20 mM Tris‐HCl [pH 7.4]) preheated to 48 °C for 20 min and washed three times in PBS. Slides were then subjected to a routine immunofluorescence staining of Mucin‐2. Finally, slides were stained with DAPI, washed with PBS, allowed to dry, and mounted in mounting medium (DAKO, S3023). The fluorescently labeled probes were listed in Table S2 (Supporting Information).

### Untargeted Metabolomics

Approximately 100 mg stool or colonic tissue was extracted with 1 mL methanol, and 60 µL 2‐Chloro‐L phenylalanine and Heptadecanoic acid was added as the internal standard. After vortexing, ultrasonic treatment and centrifugation, the supernatant was transferred into a new tube and dried. Then 60 µL 15 mg mL^−1^ methoxyamine pyridine and N,O‐Bis(trimethylsilyl)trifluoroacetamide reagent was sequentially added and incubated at 37 °C. After centrifugation, the supernatant was analyzed using the ACQUITY UPLC system (Waters Corporation, Milford, USA) coupled with AB SCIEX Triple TOF 6600 System (AB SCIEX, Framingham, MA) in positive and negative ion modes according to the manufacturer's manual. Metabolomic data were analyzed with MetaboAnalyst 5.0 (https://www.metaboanalyst.ca/MetaboAnalyst/home.xhtml).

### Gas Chromatograph Mass Spectrometry ‐Based Quantification of Linoleic Acid

0.1 g feces sample was weight and mixed with 1 mL methanol. After vortexing, ultrasonic treatment and centrifugation, the supernatant was transferred into a new tube. The pellet was further extracted with 1 mL methanol. The supernatant from the two extractions were combined and dried. Then 800 µL 0.5 mmol KOH‐methanol was added, shaked to dissolve and incubated in the dark for 1 h. 800 µL n‐hexane was further added and mixed for 1 min, and then incubated to separate layers. The upper layer of n‐hexane was transfer to a new tube, extract for 3 times. The n‐hexane phases were combined, dried and dissolved in n‐hexane, which was further analyzed using the Agilent 6890N gas chromatograph equipped with PEG‐20 M cross‐linked polyethylene glycol column.

### Colonic Lamina Propria Immune Cells Isolation

Mouse colon tissues were longitudinally cut and flushed with cold PBS to remove feces. Tissues were cut into 1‐cm pieces, followed by two round incubations in HBSS containing 5 mM EDTA, 1 mM DTT, 10 mM Hepes and 5% FBS at 37 °C for 20 min on a shaking incubator. After incubation, the suspension was filtered through a 70 µm strainer to remove the epithelial, subepithelial, and villus cells. The remaining colon pieces including lamina propria cells and muscle layer were cut into 1‐mm pieces and then incubated in 1640 medium containing 100 U mL^−1^ collagenase I (Gibco, 17 018 029), 100 U mL^−1^ collagenase IV (Gibco, 17 104 019), 50 U mL^−1^ DNase I (Roche, 0 471 672 8001) and 5% FBS for 1 h at 37 °C. After digestion, the lamina propria immune cells were enriched using a 40: 80% Percoll (GE Healthcare) density gradient centrifugation. Immune cells were recovered from the interface between the 40% and 80% Percoll layers.

### Flow Cytometry

Single‐immune cell suspensions from mouse colonic lamina propria were stained with fluorochrome‐conjugated monoclonal antibodies in PBS supplemented with 2% BSA for 30 min at 4 °C. Samples were incubated with LIVE/DEAD fixable near‐IR dead cell stain kit (Invitrogen, L34976) to stain dead cells. Samples were acquired using a BD FACSCanto II (BD bioscience) and the data was analyzed using FlowJo v10. Antibodies used were as following: anti‐CD45 (Clone 30‐F11, BioLegend, 103 133), anti‐CD4 (Clone RM4‐5, BD Pharmingen, 553 046), anti‐IL17 (Clone TC11‐18H10, BD Pharmingen, 559 502), anti‐Foxp3 (Clone PCH101, eBioscience, 17‐4776‐42).

For intracellular IFNγ detection, 1 × 10^7^ immune cells were stimulated with 20 ng mL^−1^ phorbol 12‐myristate 13‐acetate (PMA; Sigma), 1 µg mL^−1^ ionomycin (CST, 9995), and 1x brefeldin A (eBioscience, 00‐4506‐51) for 4 h at 37 °C. Cells were then washed, fixed by 2% PFA, permeabilized (BD Perm/Wash, 554 723), and stained with rat anti‐mouse IFNγ (Clone 4S.B3, eBioscience, 12‐7319‐41) or isotype control (eBioscience, 12‐4714‐82) antibody for 30 min at 4 °C according to the BD intracellular staining protocol.

### BMDM Generation

Bone marrow progenitors were harvested from femurs and tibias of WT C57BL/6 mice and cultured for 7 days with 50 ng mL^−1^ macrophage colony‐stimulating factor (M‐CSF). On day 7, media were changed to complete medium without M‐CSF and cells were treated with vehicle or 0.3 mM LA for 3 days. Then, BMDM were harvested for QPCR analysis,

### Assessment of Cell Differentiation by CD4^+^ T Cells

CD4^+^ T cells were purified from spleenocytes from WT C57BL/6 mice using EasySep Mouse CD4^+^ T Cell Isolation Kit (Stemcell, #19 852), and were further treated with individual LCFA (0.05 mM each) or conditioned media from BMDM under the condition of anti‐CD3/CD28 stimulation for 3 days. Then, the cells were collected for QPCP or flow cytometry analysis.

### Genetic Manipulation of *L. Animalis*

A deletion mutant of *L. animalis* was created by allelic replacement of LAI‐encoding gene. DNA segments (≈1 kb) upstream and downstream of the gene to be deleted were amplified by PCR with the following primers: LAI up‐forward: 5′‐ TAAAGGACCGATAACGCGCTCGAGCCACATACTACGACAACATC‐3′; LAI up‐reverse: 5′‐ GAACGGTAGATTTAAATTGTTTAAACTAGATCTGTGTCTAAATAATGA‐3′; LAI down‐forward: 5′‐ GCTATACGAACGGTACAGCCCGGGAACATATCACCCCATCTATC‐3′; and LAI down‐reverse: 5′‐ TTAGGTATGGTCGATAGTGCGTCTTCTTCCGATAATAAAGGCGAGC‐3′, and then introduced into the XhoI‐PmeI and XmaI–BbsI restriction sites of the suicide vector pNZ5319 using the method of homologous recombination, respectively. The resulting plasmid was transformed into the competent cells of *L. animalis* by electroporation (potential of 1.7 kV, capacity of 25 µF and shunt resistance of 200 Ω). Transformants were cultured on MRS plates containing 5 µg mL^−1^ chloramphenicol for 3 days. The chloramphenicol resistant transformants were selected and plated again to check for an erythromycin sensitive phenotype.

### Statistics

Data were analyzed by GraphPad Prism 7 (GraphPad Software), and statistical significance was determined by two‐tailed unpaired Student *t* test or Mann‐Whitney test. One‐way analysis of variance (ANOVA) with Dunnett's post hoc test was used for multiple comparisons. P < 0.05 was considered statistically significant.

### Ethics Approval Statement

The protocols for patient sample collection were approved by the Institutional Review Board for Clinical Research of the Shanghai Tenth People's Hospital of Tongji University (permit 2018‐KN22‐01). Written informed consent was obtained from all participants before the study protocol began. All animal studies were approved by the Institutional Animal Care and Use Committee of Tongji University (permit SHDSYY‐2018‐4157).

## Conflict of Interest

The authors declare no conflict of interest.

## Author Contributions

Y.H.Z. and F.F.S. contributed equally to this work. J.Y.L., H.L.Q., and Q.W. designed research. Y.H.Z., F.F.S., M.Y.X., Y.N.D., M.L., Y.C., L.L., and H.Y.Z. performed experiments and analyzed data. M.Q.Y., C.Q.C., J.Q.C. and C.L.M. collected human tissue samples. Y.H.Z., F.F.S., and Y.L. interpreted results of experiments and prepared Figures. Y.H.Z. and F.F.S. drafted the manuscript. J.Y.L. and H.L.Q. edited and revised the manuscript.

## Supporting information

Supporting Information

## Data Availability

The 16s rRNA data generated in this study have been deposited in the SRA database under accession codes PRJNA906015 [https://www.ncbi.nlm.nih.gov/bioproject/PRJNA906015]. The untargeted liquid chromatography‐mass spectrometry data generated in this study have been deposited in the MetaboLights database under the identifier MTBLS6618 [www.ebi.ac.uk/metabolights/MTBLS6618]. All other data needed to evaluate the conclusions of the study are present in the paper or in the supplementary materials.
