# Supplementary material for: Gastrointestinal Dysmotility Predisposes to Colitis through Regulation of Gut Microbial Composition and Linoleic Acid Metabolism
Source: Adv Sci (Weinh). 2024 Mar 13;11(20):2306297. doi: 10.1002/advs.202306297 (PMC11132037; doi:10.1002/advs.202306297)
Supplement: Supplementary file 1 — Supporting Information [file ADVS-11-2306297-s001.pdf]

ADVANCED SCIENCE
Open Access

Supporting Information

for *Adv. Sci.*, DOI 10.1002/advs.202306297

Gastrointestinal Dysmotility Predisposes to Colitis through Regulation of Gut Microbial Composition and Linoleic Acid Metabolism

Youhua Zhang, Feifei Song, Muqing Yang, Chunqiu Chen, Jiaqu Cui, Mengyu Xing, Yuna Dai, Man Li, Yuan Cao, Ling Lu, Huiyuan Zhu, Ying Liu, Chunlian Ma, Qing Wei, Huanlong Qin* and Jiyu Li**

Table S1.The baseline characteristics of patients with chronic constipation (CC), Crohn's diseases (CD), ulcerative colitis (UC), colorectal cancer (CRC) and healthy controls (HD).

	HD[#1] (n=30)	CC (n=30)	CD (n=32)	UC (n=25)	CRC[#2] (n=30)
**Age (years)**	48.62 ± 14.36	47.07 ± 17.16	33.89 ± 11.37	51.04 ± 14.72	70.40 ± 11.06
**Male/female (No.)**	16/14	17/13	25/7	18/7	20/10
**Disease duration (CD) (months)**	N/A	N/A	60.11 ± 15.56	62.08 ± 13.84	N/A
**Disease location (CD)[#3]**					
L1	N/A	N/A	7	4	N/A
L2	N/A	N/A	13	21	N/A
L3	N/A	N/A	12	0	N/A
L4	N/A	N/A	0	0	N/A
**Disease location (CRC)**					
Ascending colon	N/A	N/A	N/A	N/A	7
Transverse colon	N/A	N/A	N/A	N/A	1
Descending colon	N/A	N/A	N/A	N/A	0
Sigmoid colon	N/A	N/A	N/A	N/A	11
Rectum	N/A	N/A	N/A	N/A	11

Stool and tissue samples were collected in healthy controls, patients with CC, CD, and UC.

[#1] Only stool sample collected, used as healthy controls in gut microbiota analysis.

[#2] Only adjacent normal tissue collected, used as normal tissue control in staining and QPCR assays.

[#3] According to the Montreal classification system.

Table S2. Primers for QPCR and probes for FISH assay.

**Primers for QPCR**			
**Genes**	**Species**	**Forward primer**	**Reverse Primer**
IL-1α	Human	CCGTGAGTTTCCCAGAAGAA	ACTGCCCAAGATGAAGACCA
IL-1β	Human	ATGATGGCTTATTACAGTGGCAA	GTCGGAGATTCGTAGCTGGA
IL-8	Human	TTTTGCCAAGGAGTGCTAAAGA	AACCCTCTGCACCCAGTTTTC
TNF-α	Human	CCTCTCTCTAATCAGCCCTCTG	GAGGACCTGGGAGTAGATGAG
GAPDH	Human	CGGAGTCAACGGATTTGGTC	GACAAGCTTCCCGTTCTCAG
Tnf-α	Mouse	GAGGTTGACTTTCTCCTGGTAT	AGTGACAAGCCTGTAGCCC
Il-1β	Mouse	AAACCGCTTTTCCATCTTCTTCT	CGGCACACCCACCCTG
Il-6	Mouse	GAAGTAGGGAAGGCCGTG	CTGCAAGAGACTTCCATCCAGTT
Il-17	Mouse	GCTGAGCTTTGAGGGATGAT	CAGGGAGAGCTTCATCTGTGT
Claudin-1	Mouse	AGGTCTGGCGACATTAGTGG	CGTGGTGTTGGGTAAGAGGT
Claudin-2	Mouse	TCTACGAGGGACTGTGGATG	TCAGATTCAGCAAGGAGTCG
JAM-A	Mouse	TCTCTTCACGTCTATGATCCTGG	TTTGATGGACTCGTTCTCGGG
Occludin	Mouse	TTGAAAGTCCACCTCCTTACAGA	CCGGATAAAAAGAGTACGCTGG
ZO-1	Mouse	GCCGCTAAGAGCACAGCAA	TCCCCACTCTGAAAATGAGGA
Gapdh	Mouse	CCTGTTGCTGTAGCCGTATTCA	CCAGGTTGTCTCCTGCGACTT
Bacteria		TCCTACGGGAGGCAGCAGT	GGACTACCAGGGTATCTATCCTGTT
Lactobacillus		TGGAAACAGRTGCTAATACCG	GTCCATTGTGGAAGATTCCC
L. animalis		CTTGCACTCACCGATAAAGAG	GTCCATTGTGGAAGATTCCC
L. johnsonii		GAGCTTGCCTAGATGATTTTAG	GTCCATTGTGGAAGATTCCC
A. muciniphila		AAGGTGGGGACTCTGGCGAG	CTTGCGGTTGGCTTCAGATAC
**Probes for FISH**			
**Name**	**Species**	**Sequence**	**Modification**
Lacb0722	*Lactobacillus (genus)*	YCACCGCTACACATGRAGTTCCACT	5'FAM
Muc-1437	*A. muciniphila*	CCTTGCGGTTGGCTTCAGAT	5'Cy3

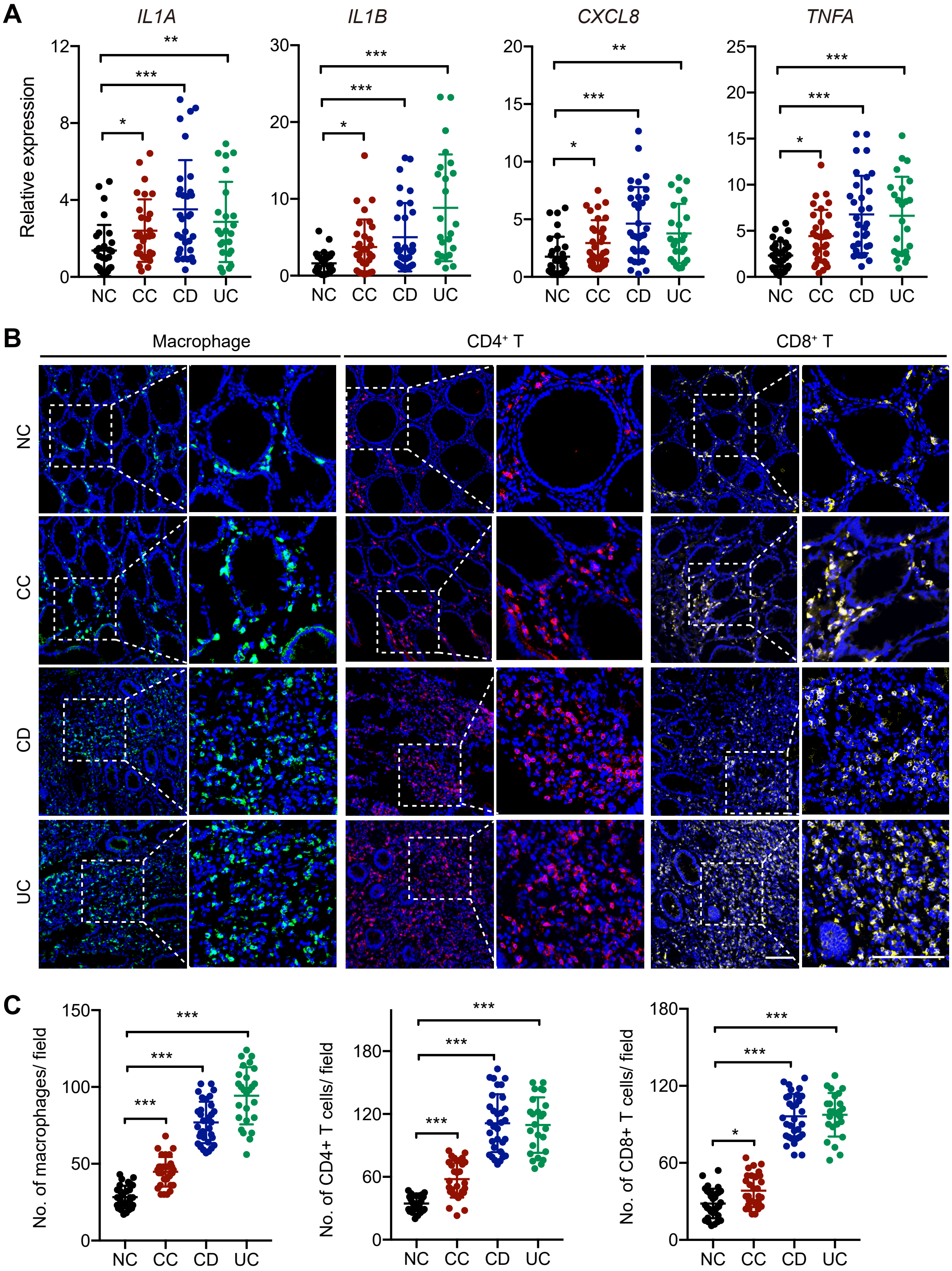

Figure S1. Colonic musoca from patients with chronic constipation displayed increased mucosal inflammation. A) QPCR analysis of inflammatory gene expression in colonic tissues from patients with chronic constipation (CC) (n=30), inflamed region from patients with Crohn's disease (CD) (n=32), inflamed region from patients with ulcerative colitis (UC) (n=25), and normal adjacent tissue from patients with colorectal cancer served as normal control (NC) (n=30). B) Immunofluorescence staining of macrophages, $CD4^{+}$ T and $CD8^{+}$ T cells in tissues displayed in A. Scale bars, 100 μm. C) Quantification of macrophages, $CD4^{+}$ T and $CD8^{+}$ T cells per field in B. In A and C, data represent mean ± SD; $^{*}P < 0.05$, $^{**}P < 0.01$, $^{***}P < 0.001$ by one-way ANOVA with Dunnett's post hoc test.

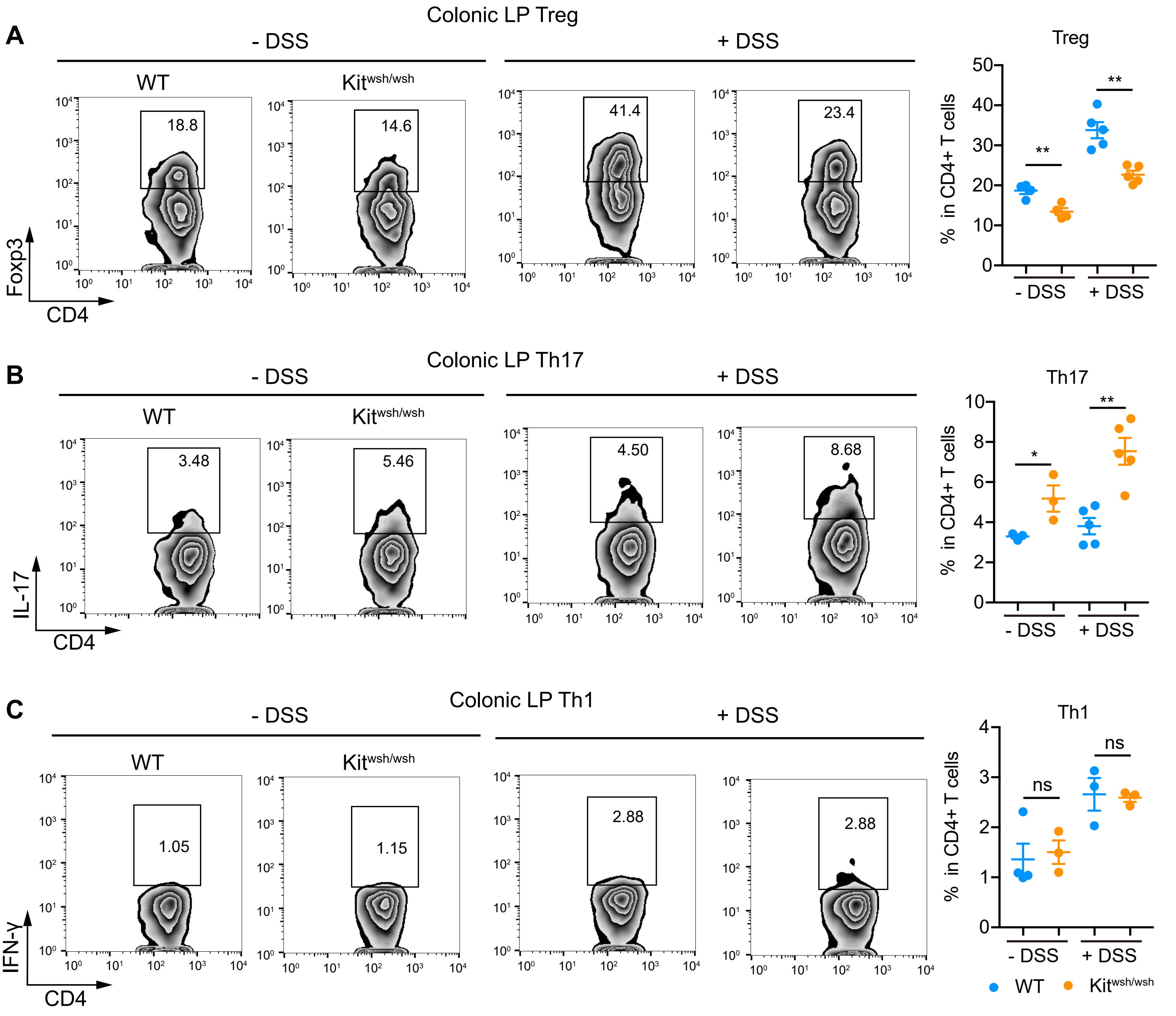

Figure S2. **DSS treated Kit$^{wsh/wsh}$ mice displayed altered infiltraiton of Treg and Th17**. The abundance of Treg (A), Th17 (B), Th1 (C) cells in colonic lamina propria (LP) were analyzed by flow cytometry. Data represent mean ± SEM; ns, not significant, *P < 0.05, **P < 0.01 by unpaired Student's *t* test.

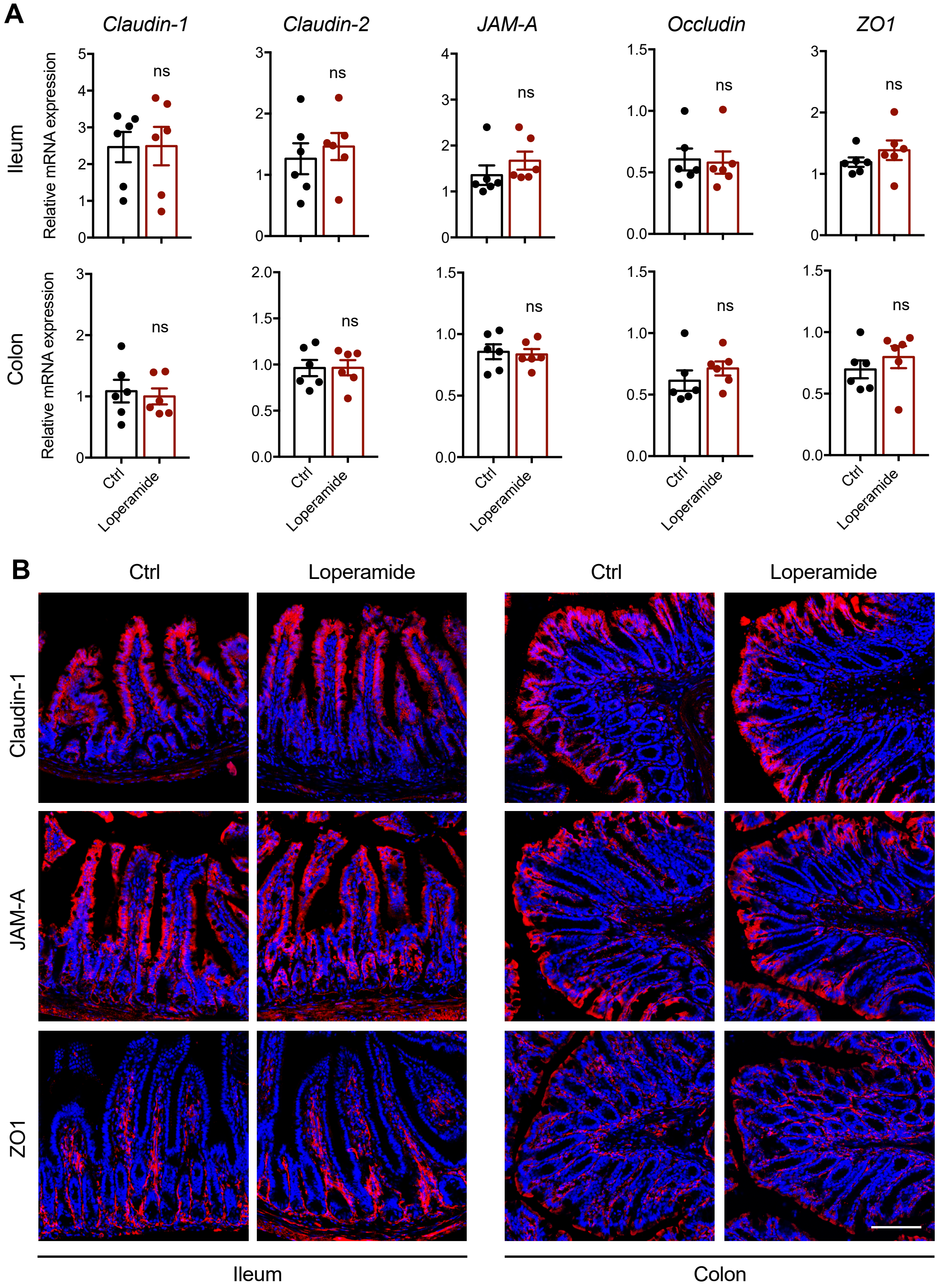

Figure S3. Loperamide treatment did not alter the expression of tight junction proteins in intestinal and colonic tissues. WT mice (n=6/group) were gavaged with 10 mg/kg body weight (b.w.) loperamide every day for 7 days. The expression of tight junction proteins including *claudin-1*, *claudin-2*, *JAM-A*, *occludin*, and *ZO-1* was examined by QPCR (A) and immunofluorescence staining (B). Scale bar, 100 µm. Data represent means ± SEM; ns, not significant by two-sided Student *t* test.

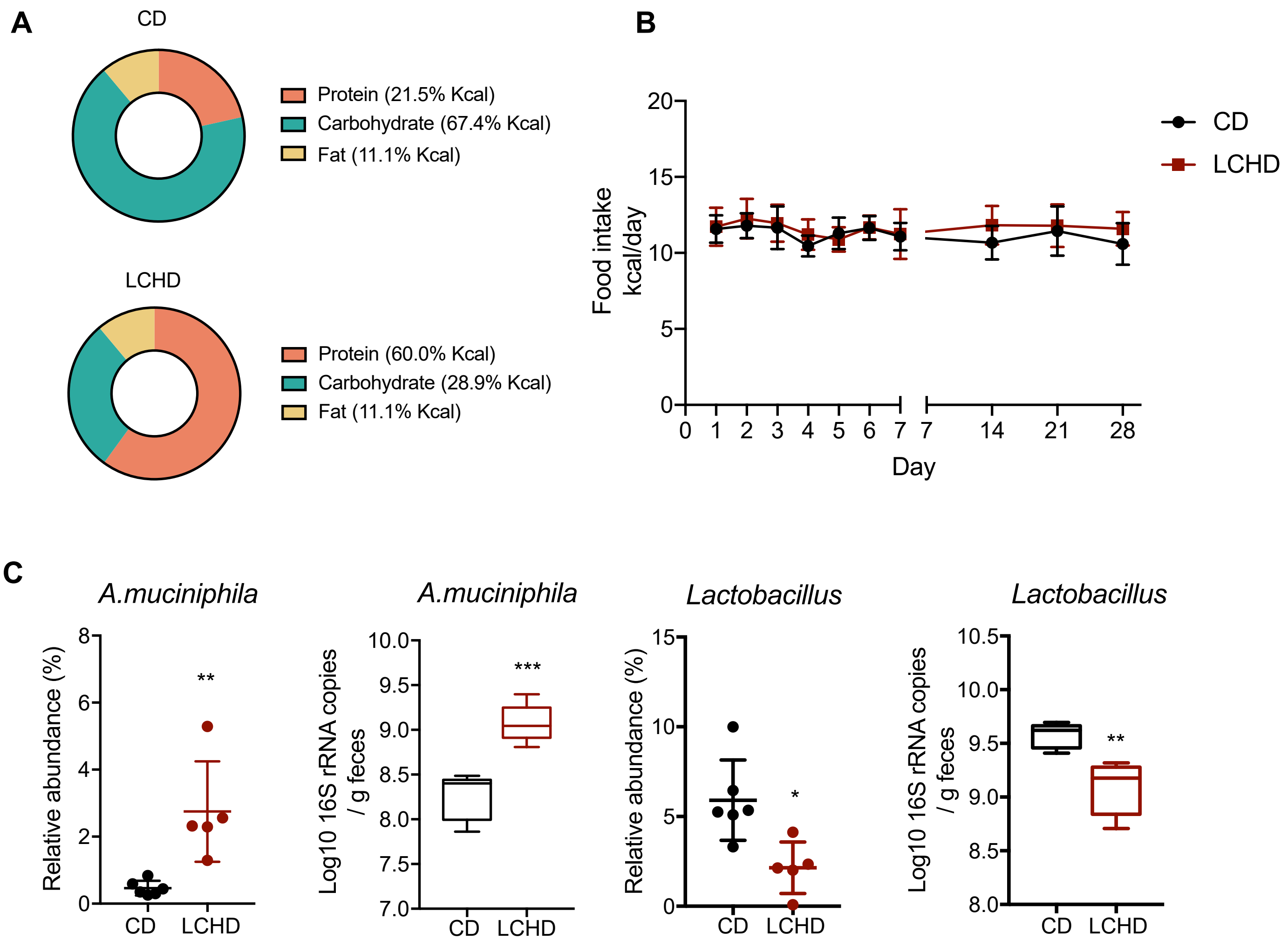

Figure S4. Mice fed with low-carbohydrate and high-protein diet displayed an elevated abundance of *A. muciniphila*, while decreased abundance of *Lactobacillus*. A) Composition of the control diet (CD) and the low-carbohydrate and high-protein diet (LCHD) represented in percent calories. B) Food intake of mice fed with CD or LCHD. C) QPCR analysis of the abundance of *A. muciniphila* and *Lactobacillus* in fecal sample from mice fed with CD or LCHD for 4 weeks. Data represent mean ± SD; $*P < 0.05$, $**P < 0.01$, $***P < 0.001$, ns, not significant by two-sided Student *t* test.

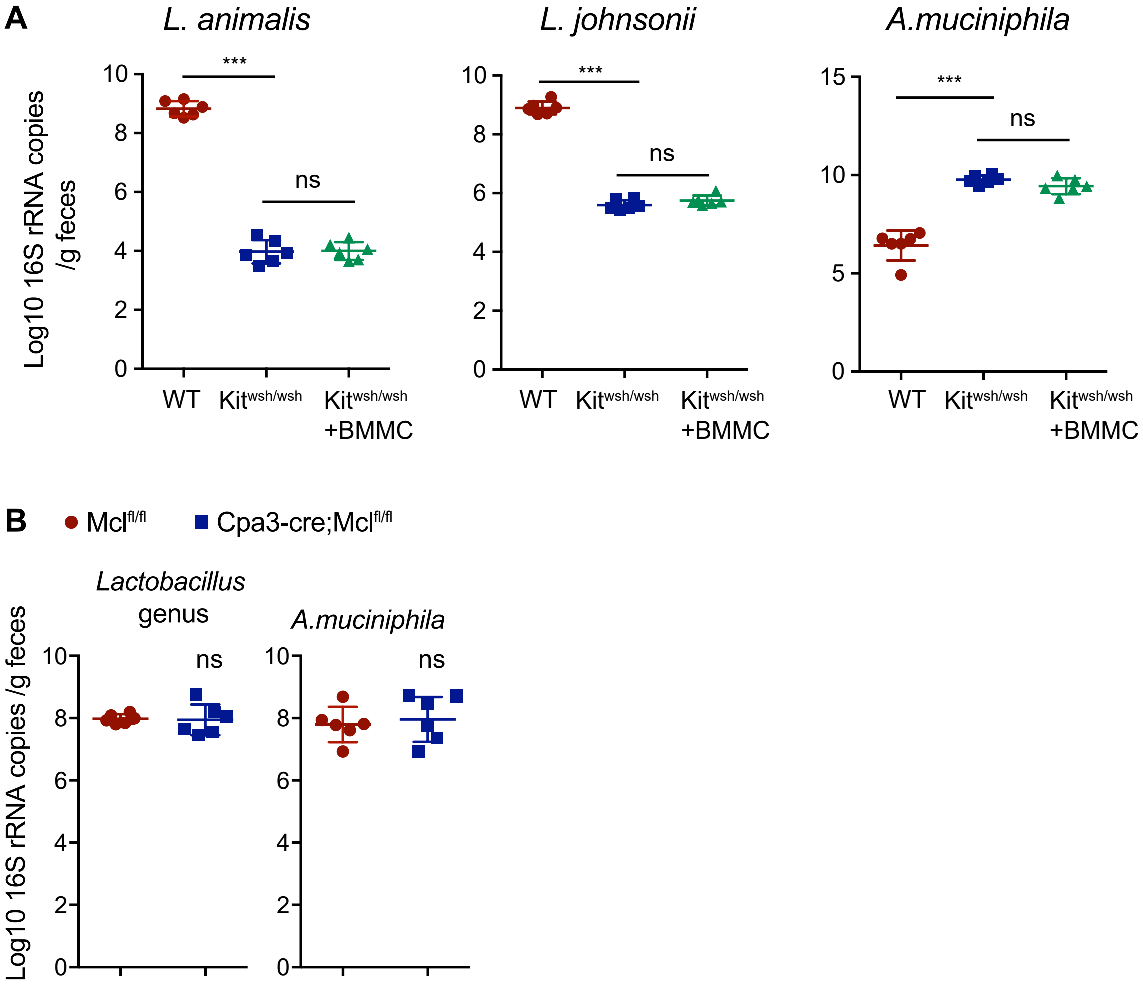

Figure S5. GI dysmotility was responsible for the altered gut microbiota in Kit$^{wsh/wsh}$ mice, rather than mast cell. A) QPCR analysis of the absolute abundance of *L. animalis*, *L. johnsonii* and *A. muciniphila* in feces of WT, Kit$^{wsh/wsh}$ and Kit$^{wsh/wsh}$ mice reconstituted with bone marrow derived mast cells (BMMC) (n=6/group). B) QPCR analysis of the absolute abundance of *Lactobacillus* genus and *A. muciniphila* in feces of Mcl$^{fl/fl}$ and Cpa3-cre; Mcl$^{fl/fl}$ mice (n=6/group). Data represent means ± SEM; ns, not significant, ***$P < 0.001$ by one-way ANOVA with Dunnett's post hoc test (A) and unpaired Student's *t* test (B).

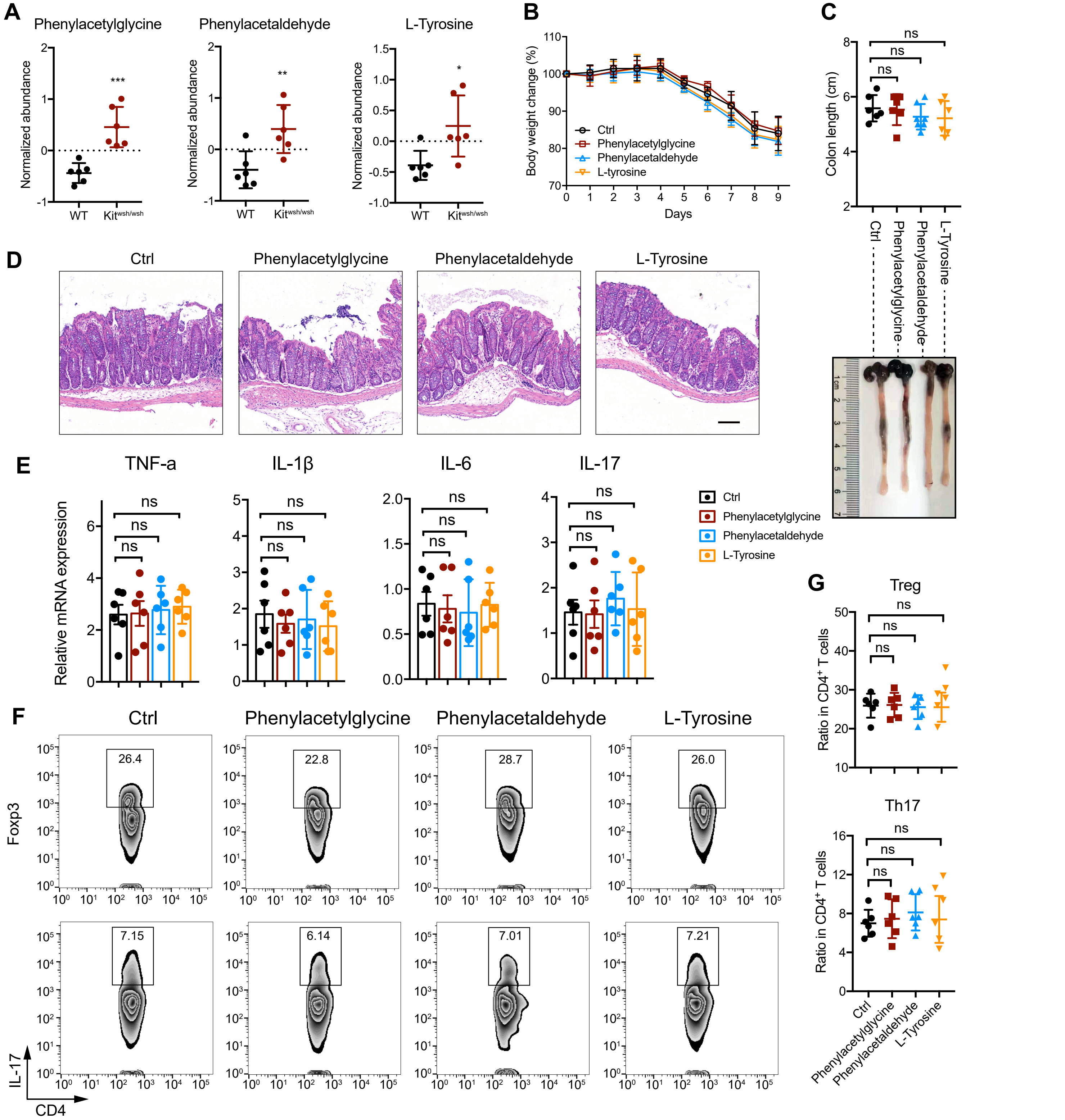

Figure S6. Phenylalanine metabolism did not significantly alter susceptibility to colitis. A) The normalized abundance of metabolites enriched in phenylalanine metabolism which includes phenylacetylglycine, phenylacetaldehyde, and L-Tyrosine in WT and Kit$^{wsh/wsh}$ mice. B-G) WT mice (n=6/group) were treated with phenylacetylglycine, phenylacetaldehyde, and L-Tyrosine in drinking water at a concentration of 200 mg/kg per day for 2 weeks, followed by a 2.5% DSS treatment for 7 days and water 2 days. The body weight changes (B), colon lengths (C), representative images of H&E-stained colon sections (D), proinflammatory cytokine expression (E) and infiltration of Treg and Th17 in colonic tissue (F, G) were monitored or analyzed. Scale bar (D), 100 μm. In A-C, E, and G, data represent mean ± SD; *P < 0.05, **P < 0.01, ***P < 0.001, ns, not significant by one-way ANOVA with Dunnett's post hoc test.

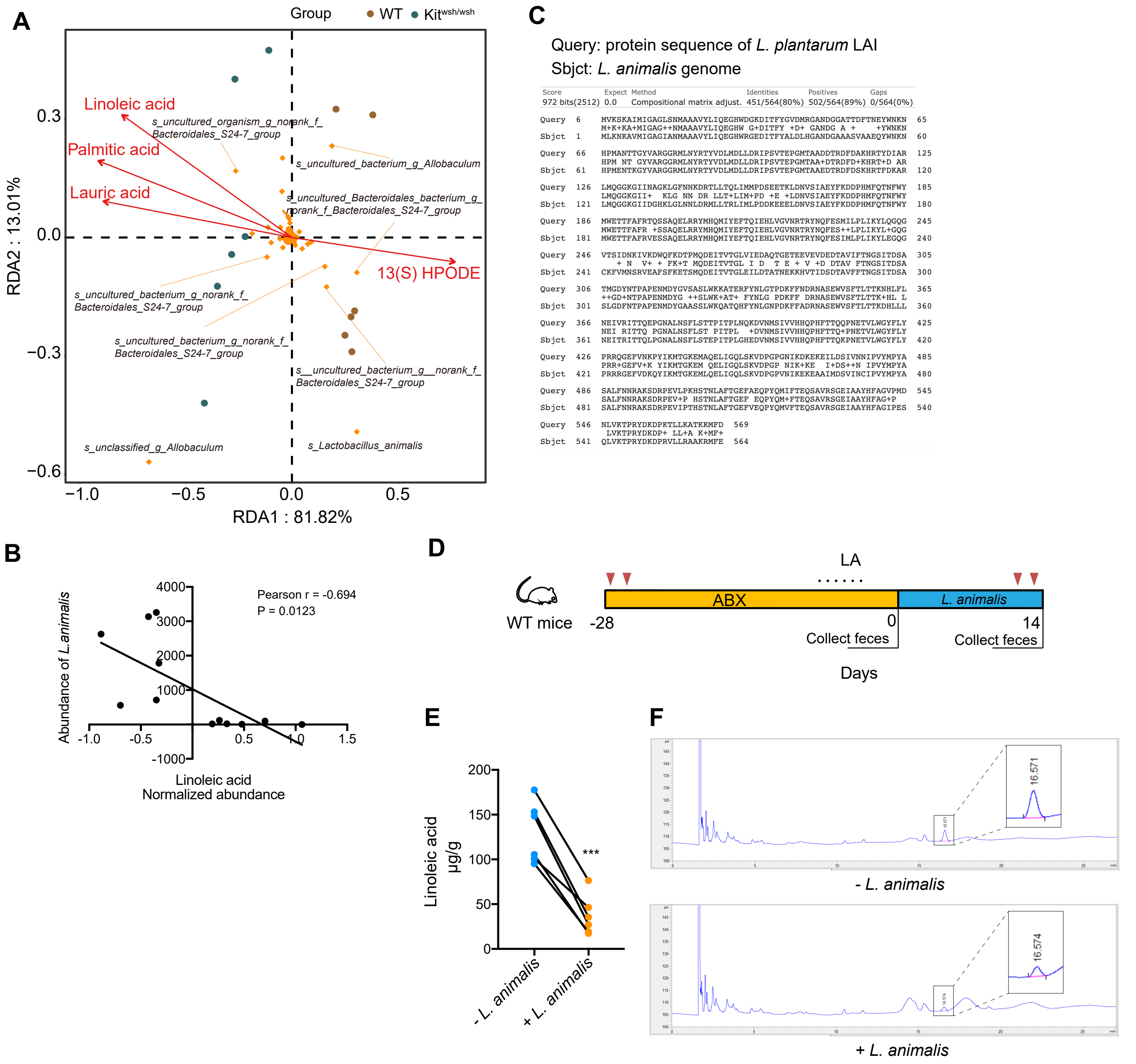

Figure S7. ***L. animalis* has the ability to convert linoleic acid.** A) Redundancy analysis (RDA) of species that different between Kit$^{wsh/wsh}$ and WT mice, and long chain fatty acids. Only top 20 different bacterial species were labeled. B) Correlation analysis of *L. animalis* and fecal levels of linoleic acid. C) BLAST search of the LAI homologous enzyme in *L. animalis* genome. D-F) WT mice were treated with a cocktail of antibiotics (ABX) for 4 weeks, and then gavaged with *L. animalis* for 2 weeks. Linoleic acid (LA) was administrated during the whole process. Fecal samples were collected and the amount of LA was quantified by gas chromatography before and after *L. animalis* gavage. The quantification data was shown in E and the representative chromatogram was shown in F. ***P < 0.001 by paired two-sided Student *t* test.

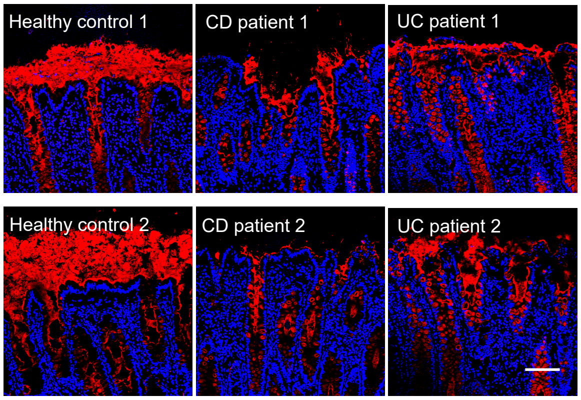

Figure S8. Representative images of mucus layer stained using anti-Mucin2 antibody in adjacent normal tissue from CRC patients and inflamed colon tissue from CD and UC patients. Scale bar, 100 μm.
